# Quantum-mechanical analysis of effect of alloying elements on *ε*-martensite start temperature of steels

**DOI:** 10.1038/s41598-017-18230-z

**Published:** 2017-12-19

**Authors:** J. H. Jang, J. Moon, H.-Y. Ha, T.-H. Lee, D.-W. Suh

**Affiliations:** 1 Ferrous Alloy Department, Korea Institute of Materials Science, Changwon, 51508 Republic of Korea; 2 Graduate Institute of Ferrous Technology, POSTECH, Pohang, 37673 Republic of Korea

## Abstract

With regard to the transformation mechanism of austenitic high manganese steel, the prediction of the *ε*-martensite start temperature is a critical consideration in alloy design. Evaluation of the *ε*-martensite start temperature makes it possible to predict the microstructure and to understand the phase transformation occurring during deformation. Here we use the quantum mechanical calculation of random alloys to understand the physics for *ε*-martensitic transformation in steels. We could find the linear relationship between the measured *ε*-martensite start temperatures and the crystal structure stability for various compositions. We also could estimate the effect of several alloying elements. It is expected that the effect of decreasing the temperatures for the same amount of alloying elements addition will be larger moving farther from Group VIII. By creating a free-energy model that reflects the temperature effect, we were able to calculate the average driving force required for the *ε*-martensitic transformations.

## Introduction

Martensitic transformation in steels is an important phenomenon that affects their mechanical properties and has been studied extensively. In particular, as an ongoing demand for the development of transformation-induced plasticity (TRIP) and twinning-induced plasticity (TWIP) steels that offer excellent combination of high strength and high ductility, understanding the martensitic transformation phenomenon has received lots of attention. Accurate prediction of the martensite-start (*M*_*s*_) temperature has been central issue in understanding the nature of martensitic transformation, and it can be used to design alloys and heat treatments to achieve the desired properties and microstructure.

The martensitic transformation is a process that changes a crystal structure by a homogeneous deformation without any change in composition. This transformation is initiated when the difference in free energy between two crystal structures exceeds a certain critical value, which is determined by stored energy or kinetic phenomena. The basic theory for predicting the *M*_*s*_ temperature was well established by Kaufmann, Cohen, and Olson *et al*.^1,2^. In steel, the initial austenite (*γ*) phase of the face-centered cubic (FCC) structure is mainly transformed into *α*′-martensite of the body-centered tetragonal (BCT) structure. The free energy change accompanying *α*′-martensite formation at the *M*_*S*_ temperature of steel is between −900 and −1400 J/mol as a function of the carbon content^3^. The *M*_*S*_ temperature of *α*′-martensite (${M}_{S}^{\alpha ^{\prime} }$) can therefore be correlated well with thermodynamic database of the relevant components, along with precise experimental data^3–6^. The ${M}_{S}^{\alpha ^{\prime} }$ temperature change caused by a new alloying element whose influence has not been confirmed yet, is predictable when the free energy difference is calculated.

Recently, austenitic steels including high Mn steels, light-weight steels, austenitic stainless steels, and shape-memory alloys have been studied extensively^7–9^. In those alloys, a change from austenite to *ε*-martensite with a hexagonal close packed (HCP) structure rather than *α*′-martensite may be observed during cooling^9^. It is generally accepted that the ordinary deformation mechanism of austenitic steel is mainly determined by the stacking fault energy (SFE), and that the main factor determining SFE is the relative lattice stabilities of the FCC and HCP structures, as in the *ε*-martensitic transformation^10^. Therefore, in the alloy design and process optimization, predicting the *M*_*S*_ temperature of *ε*-martensite (${M}_{S}^{{\rm{\varepsilon }}}$) for each composition is directly related to understanding the compositional dependence on the SFE. In previous studies, attempts have been made to predict the effect of the allying elements on the ${M}_{S}^{{\rm{\varepsilon }}}$ temperature based on the thermodynamic driving force^11–13^. However, unlike ${M}_{S}^{\alpha ^{\prime} }$ temperature, the prediction of the ${M}_{S}^{{\rm{\varepsilon }}}$ temperature is limited by a lack of existing databases or measurement data. Although first-principles studies on the effect of elements on the lattice stability or SFE are ongoing, the compositional range of the alloys remain limited^14–16^.

The main difficulty in the application of first-principles calculations to structural materials, such as steels, is related to the presence of various kinds of disorder, including chemical and magnetic disorder. To illustrate the energetics of these fully or partially disordered systems, various techniques have been developed. The most direct method is to construct a sufficiently large supercell containing many atoms. This is used to determine the energy by constructing disorder for the various atoms within it. However, this is not practical because it requires a lot of computation for very large systems. To solve this problem, a virtual crystal approximation (VCA) method^17–19^, cluster expansion (CE) formalism^20^, and special quasi-random structure (SQS)^21^ method have been utilized. These methods are less accurate in predicting the properties of alloys, or have only limited applicability to alloys, because a large amount of calculation is required when calculating multi-component alloys. On the other hand, the coherent potential approximation (CPA) method can efficiently simulate multi-component substitutional disorder^22,23^. This approximation was introduced by Soven and Taylor, and formulated within the framework of multiple-scattering theory, using the Green function formalism^24–26^. The CPA has been applied to the exact muffin-tin orbital (EMTO) method and has successfully predicted the properties of various alloys^27–31^.

Here we consider the compositional dependence on the ${M}_{S}^{{\rm{\varepsilon }}}$ temperature by collecting a large number of data sources, which are combinations of the chemical composition and the ${M}_{S}^{{\rm{\varepsilon }}}$ temperature from the literatures. To determine the composition-${M}_{S}^{{\rm{\varepsilon }}}$ temperature relations, we used multiple-linear regression and quantum-mechanical calculations, within the framework of the EMTO-CPA method. We find that there is a close linear relationship between the ${M}_{S}^{{\rm{\varepsilon }}}$ temperature and the crystal structure stability of the anti-ferromagnetic FCC as well as the paramagnetic HCP at the 0 K. Therefore, the effect of alloying elements on ${M}_{S}^{{\rm{\varepsilon }}}$ temperature can be estimated. Phosphorous is expected to promote *ε*-martensitic transformation and to increase the ${M}_{S}^{{\rm{\varepsilon }}}$ temperature. In the case of transition metals, the effect of suppressing *ε*-martensitic transformation is further enhanced as the distance from Group VIII is increased. Based on the measured *T*_0_ and ${M}_{S}^{{\rm{\varepsilon }}}$ temperature, the force driving the transformation to *ε*-martensite was calculated to be −0.281 kJ mol^−1^ on average. The findings enable to predict the effect of alloying elements on the microstructure and phase transformation occurring during deformation in steels.

## Results and Discussions

### Multiple linear regression model approach

**Figure 1:**
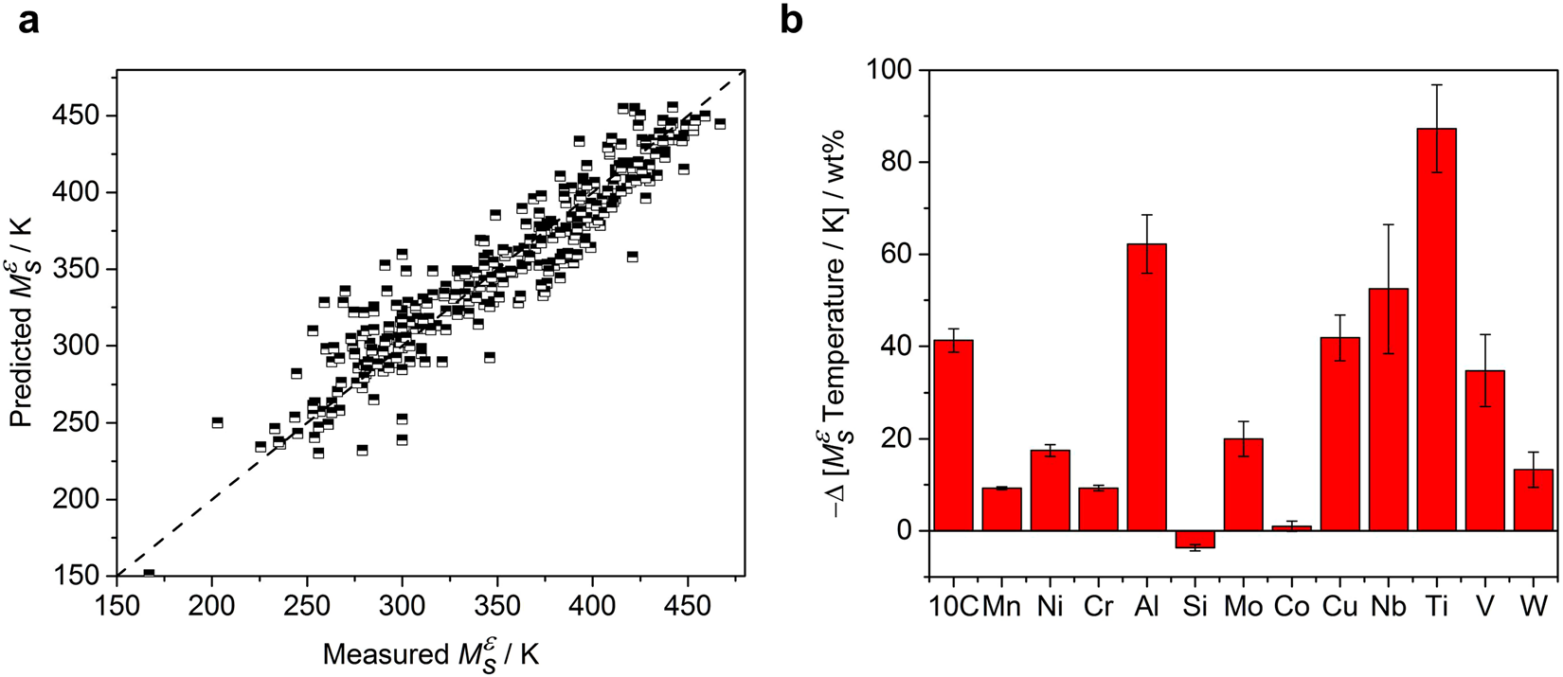
Multiple linear regression analysis results for ${M}_{S}^{{\rm{\varepsilon }}}$ temperature. (**a)** The estimated ${M}_{S}^{{\rm{\varepsilon }}}$ temperatures versus measured values. (**b)** The regression coefficients which represent the contribution of each element to the change of the ${M}_{S}^{{\rm{\varepsilon }}}$ temperature. The error bars correspond to the standard error of each coefficient. In the case of carbon, the coefficient was divided by 10 to allow display on the same scale.

The multiple linear regression model for ${M}_{S}^{{\rm{\varepsilon }}}$ temperature was constructed using the least square fit method with 13 input parameters corresponding to the amount of each element added based on 322 data sets. The statistics of the collected data are summarized in Table 1, and the results of the multiple linear regression analysis is as follows,

1 $$\begin{array}{rcl}{M}_{S}^{\varepsilon }/K & = & \mathrm{(582.1}\pm \mathrm{6.6)}-\mathrm{(413.2}\pm \mathrm{24.9)}{w}_{{\rm{C}}}-\mathrm{(9.3}\pm \mathrm{0.3)}{w}_{{\rm{Mn}}}\\  &  & -\,\mathrm{(17.5}\pm \mathrm{1.3)}{w}_{{\rm{Ni}}}-\mathrm{(9.3}\pm \mathrm{0.6)}{w}_{{\rm{Cr}}}-\mathrm{(62.2}\pm \mathrm{6.3)}{w}_{{\rm{Al}}}\\  &  & +\,\mathrm{(3.7}\pm \mathrm{0.7)}{w}_{{\rm{Si}}}-\mathrm{(20.0}\pm \mathrm{3.8)}{w}_{{\rm{Mo}}}-\mathrm{(1.0}\pm \mathrm{1.1)}{w}_{{\rm{Co}}}\\  &  & -\,\mathrm{(41.9}\pm \mathrm{4.9)}{w}_{{\rm{Cu}}}-\mathrm{(52.5}\pm \mathrm{14.0)}{w}_{{\rm{Nb}}}-\mathrm{(87.3}\pm \mathrm{9.5)}{w}_{{\rm{Ti}}}\\  &  & -\,\mathrm{(34.8}\pm \mathrm{7.8)}{w}_{{\rm{V}}}-\mathrm{(13.3}\pm \mathrm{3.8)}{w}_{{\rm{W}}}\end{array}$$

where *w*_*M*_ is the amount of alloying element added, with wt% for M = C, Mn, Ni, Cr, Al, Si, Mo, Co, Cu, Nb, Ti, V, and W. The correlation-coefficient (*R*) of the model was 0.93, and the squared correlation-coefficient (*R* ^2^), which indicates the ratio of the variability of the response explained by the model, was 0.87. Figure 1 shows the results, including predicted values and coefficients for each input variables, according to the model. This model can be correlated well with the ${M}_{S}^{{\rm{\varepsilon }}}$ temperature depending on the alloying elements, but there are some limitations. (1) It cannot exhibit a non-linear relationship and cannot justify the dependencies between the variables. (2) This model cannot separate the effects of the alloying elements on the ${M}_{S}^{{\rm{\varepsilon }}}$ temperature and other effects, such as the initial austenite grain size and errors due to the measurement methods. (3) The effects of other alloying elements (e. g. phosphorus, sulfur, silver and gold) that were not included in model construction cannot be explored.

**Table 1:** Collected data statistics on *ε*-martensite start temperature.

	Minimum	Maximum	Average	Standard deviation
C	0	0.35	0.02	0.05
Mn	11.2	35.9	22.2	6
Ni	0	6.8	0.48	1.46
Cr	0	13.7	1.53	3.20
Al	0	2.22	0.03	0.19
Si	0	7.12	2.08	2.52
Mo	0	4.46	0.03	0.31
Co	0	8.0	0.30	1.17
Cu	0	3.08	0.03	0.25
Nb	0	1.21	0.01	0.09
Ti	0	1.72	0.01	0.13
V	0	2.2	0.01	0.15
W	0	4.48	0.03	0.31
${M}_{S}^{{\rm{\varepsilon }}}$/K	167	467	350.49	59.60

Minimum, maximum, average and standard deviation for 322 individual measurements for the ${M}_{S}^{{\rm{\varepsilon }}}$ temperatures. The values are rounded off to significant figures after the decimal point. The concentration of each solute is wt%, and the units of measured ${M}_{S}^{{\rm{\varepsilon }}}$ temperature is K.

### Thermodynamic driving forces using the CALPHAD approach

**Figure 2:**
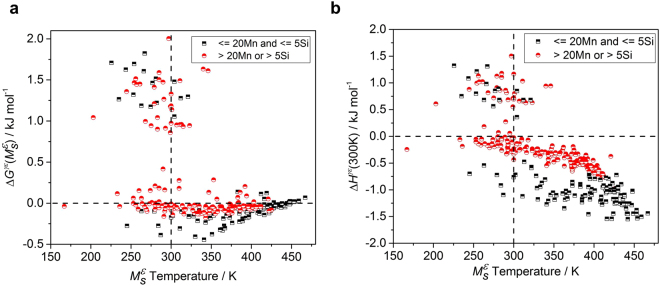
Thermodynamic quantities calculated using the TCFE 7.0 database and ThermoCalc as a function of the measured ${M}_{S}^{{\rm{\varepsilon }}}$ temperature. The half-black squares represent a composition containing up to 20 wt% Mn and 5 wt% Si, the range supported by the thermodynamic database, and the half-red dots indicate data corresponding to other compositions. (**a**) The calculated free energy change at the measured ${M}_{S}^{{\rm{\varepsilon }}}$ temperature. (**b**) The calculated enthalpy change at 300 K.

The stability of austenite and *ε*-martensite during cooling is dependent on Δ*G* ^*γε*^(*T*), the Gibbs free energy difference between FCC and HCP structure at a given temperature (T). Here, thermodynamic quantities were evaluated based on the TCFE7.0 database^32^, and they were summarized in Fig. 2. Figure 2a and b show the calculated free energy differences at ${M}_{S}^{{\rm{\varepsilon }}}$ temperature and enthalpy differences at 300 K between the FCC and HCP structures, respectively. We calculated the enthalpy difference at 300 K, which is the lower limit of supported range of the database. The results of the free energy difference showed a wide range (−0.5 to +2.0 kJ mol^−1^) for both the part supported by the database and the part unsupported over the composition range. A lot of data show very low free energy changes near zero, and some data even have positive values. This is unreasonable considering the driving force required by the accompanying strain energy during martensitic transformation, thus limiting the prediction of the ${M}_{S}^{{\rm{\varepsilon }}}$ temperature^12^. There are 53 data corresponding to ${\rm{\Delta }}{G}^{\gamma \varepsilon }\mathrm{ > 0.5}$ kJ mol^−1^, which means that austenite is more stable compared to *ε*-martensite. Included were corresponding data on 4.6–11.6 wt% Cr and 4.2–7.03 wt% Si, with C, Mn, and Ni. Adding large amounts of Cr and Si result in a large Δ*G* ^*γε*^ value, so the thermodynamic data corresponding to this concentration is relatively unreliable. When comparing the stability of the two structures through enthalpy difference shown in Fig. 2b, we could not find any relationship between the calculated data and ${M}_{S}^{{\rm{\varepsilon }}}$ temperature. It is believed that the thermodynamic database might be less accurate than the experimental error in measuring the ${M}_{S}^{{\rm{\varepsilon }}}$ temperature, since the distribution of thermodynamic quantities is not normal.

### Quantum mechanical calculation for lattice stability

Using the calculated total energies from quantum-mechanical calculations, we could determine the effect of alloying elements on the lattice stability based on the following equation.

2 $${\rm{\Delta }}{H}^{\gamma {\rm{\varepsilon }}}\mathrm{(0}\,{\rm{K}})={E}_{tot}^{\varepsilon }-{E}_{tot}^{\gamma }$$

where ${E}_{tot}^{{\rm{\varepsilon }}}$ and ${E}_{tot}^{\gamma }$ represent the equilibrium total energy per atomic site for the paramagnetic HCP and anti-ferromagnetic FCC structures, respectively. To calculate the total energy of the FCC and HCP structures, the CPA method was applied to reflect the disordering of the alloys. It is important to note that the CPA method has shortcomings that it cannot include the effect of the inhomogeneous distributions and short-range ordering of alloying elements because it uses single site approximation to simulate random alloys. Since the *ε*-martensitic transformation is a diffusionless transformation from austenite at high temperature, it is believed that the effect of inhomogeneous distribution of alloying elements on the crystal structure stability is not significant.

The characteristics of *ε*-martensitic transformations of high Mn steels are largely associated to their magnetic properties, and the role of anti-ferromagneitsm has been carefully analyzed in several studies^33–35^. The austenite and *ε*-martensite are stable at high temperatures in a paramagnetic state, but a magnetic transition occurs in an anti-ferromagnetic state as they fall below their respective Néel temperatures (*T*_*N*_(*γ*) and *T*_*N*_(*ε*)). Experimental measurements show that *ε*-martensite is anti-ferromagnetic in some Fe-X systems, but its *T*_*N*_(*ε*) is approximately 230 K, which is below the region of interest in the *ε*-martensitic transformations^34,35^. In contrast, the parent austenite is strongly stabilized by anti-ferromagnetic ordering below 500 K in a Fe-Mn binary system^35^. Most of the ${M}_{S}^{{\rm{\varepsilon }}}$ temperature data collected in this study are in the range of 230–460 K, and anti-ferromagnetic FCC and paramagnetic HCP are regarded as stable in this temperature range. It is therefore reasonable to compare the ${M}_{S}^{{\rm{\varepsilon }}}$ temperature with the crystal structure stability based on these magnetic state. One aspect to keep in mind is that other elements besides Fe and Mn affect *T*_*N*_(*γ*), at which the magnetic structure of the parent austenite is changed during the *ε*-martensitic transformation^36^. This is likely to be one of the main causes of the prediction error of the following model.

**Figure 3:**
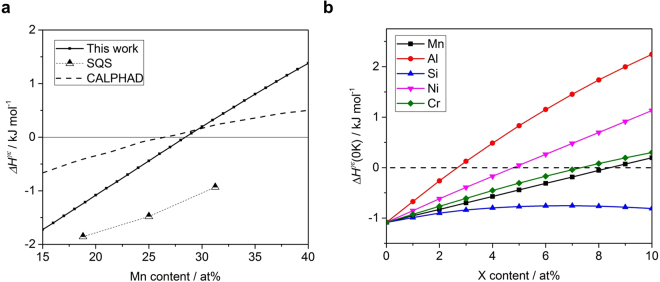
Alloying effect on lattice stability. Calculated zero temperature enthalpy difference Δ*H* ^*γε*^ as a function of alloying element additions. (**a)** For Fe-Mn alloys, enthalpy differences between HCP and FCC calculated in this study (solid black line), special quasi random structure (SQS) method^16^, and CALPHAD method^15^. (**b**) For Fe-20Mn-X at% alloys, enthalpy difference according to X = Mn (black squares), Al (red dots), Si (blue upper triangles), Ni (purple arrows) and Cr (green diamonds).

The Δ*H* ^*γε*^(0 K) increased with the addition of Mn from −1.72 kJ mol^−1^ at 15 at% to 1.48 kJ mol^−1^ at 40 at% as shown in Fig. 3a. At a composition of more than 28 at%, the lattice stabilities of the FCC and HCP structures reversed, which is similar to the results predicted for 26 at% by the existing CALPHAD calculations^15^. The compositional dependence of Δ*H* ^*γε*^(0 K) in the Fe-20Mn-X at% system including Al, Si, Ni, and Cr are shown in Fig. 3b. When the same amount of each element was added, Al had the greatest effect on increasing the austenite stability, and was effective in the order of Ni, Cr, Mn, and Si. Although, the effect of increasing the stability was mostly linear, non-linear relationship between Si content and lattice stability was obtained; the value of Si was highest at around 7 at% and further addition of Si decreased the stability of austenite. This is consistent with the effect of Si on SFE, which shows the highest effect at about 3–4 wt%^37^.

**Figure 4:**
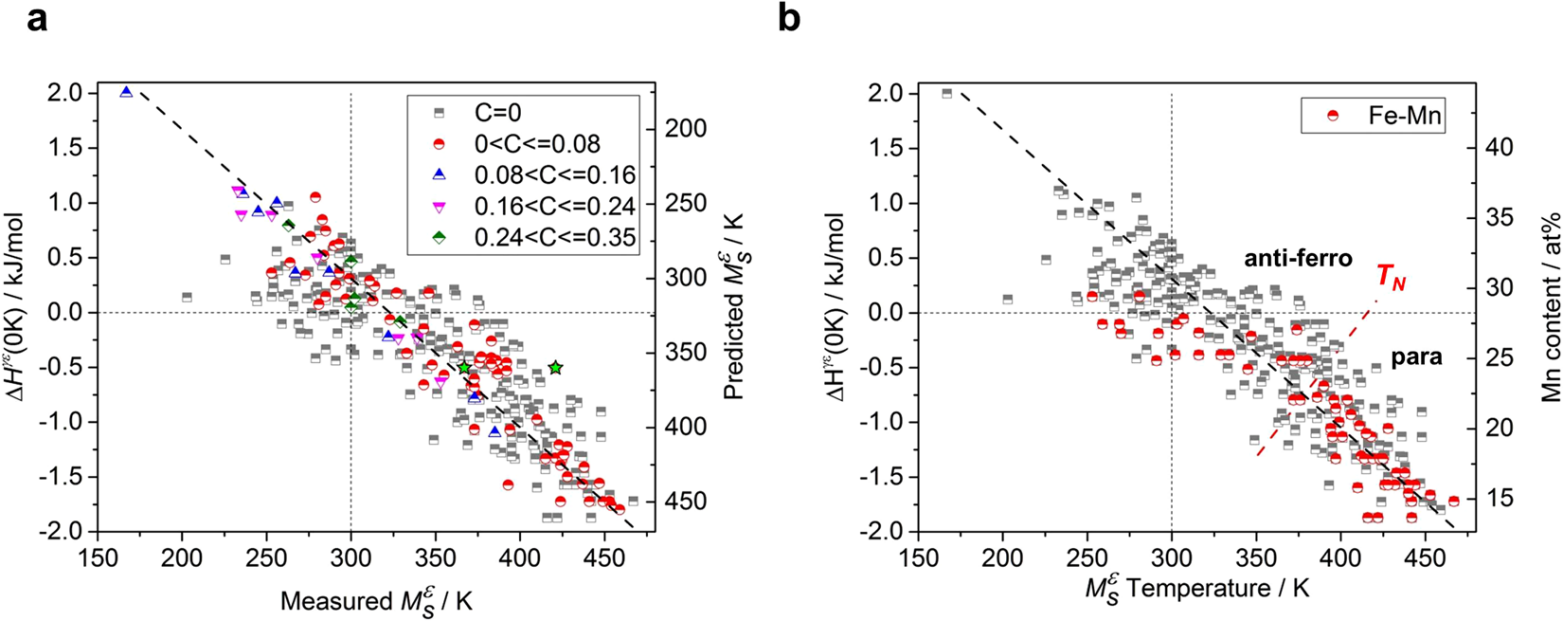
Relationship between ${M}_{S}^{{\rm{\varepsilon }}}$ temperature and lattice stability. Calculated zero temperature enthalpy difference between HCP and FCC, Δ*H* ^*γε*^(0 K), versus ${M}_{S}^{{\rm{\varepsilon }}}$ temperature: (**a)**, Grouped according to C amount (no carbon: half-grey squares, 0–0.08 wt%: half-red dots, 0.08–0.16 wt%: part-blue triangles, 0.16–0.24 wt%: part-purple triangles, 0.24–0.35 wt%: half-green diamonds), The green stars represent data corresponding to Fe-0.0017C-24.03Mn wt%. The right axis shows the ${M}_{S}^{{\rm{\varepsilon }}}$ temperature predicted by Eq. (3). (**b)** Classification of Fe-Mn binary alloys (half-red dots). The right axis represents the amount of Mn corresponding to Δ*H* ^*γε*^(0 K) for Fe-Mn binary alloys. *T*_*N*_ is the Néel temperature of austenite for the Fe-Mn binary alloy^62^.

The Δ*H* ^*γε*^(0 K) values for alloys against measured ${M}_{S}^{{\rm{\varepsilon }}}$ temperature are represented in Fig. 4. Overall, the lower Δ*H* ^*γε*^(0 K) was, the higher the stability of HCP, was obtained, which corresponded to inverse correlationship, the higher ${M}_{S}^{{\rm{\varepsilon }}}$ temperature. The following relation was obtained based on linear regression models

3 $${M}_{S}^{{\rm{\varepsilon }}}/{\rm{K}}=322.85-73.54\times {\rm{\Delta }}{H}^{\gamma \varepsilon }\mathrm{(0}\,{\rm{K}})/{\rm{kJ}}\,{{\rm{mol}}}^{-1}$$,

and

4 $${\rm{\Delta }}{H}^{\gamma \varepsilon }\mathrm{(0}\,{\rm{K}})/{\rm{kJ}}\,{{\rm{mol}}}^{-1}=4.390-0.0136\times {M}_{S}^{\varepsilon }/{\rm{K}}.$$

This model has a close relationship with *R*(0.87) and *R* ^2^(0.77), taking into account the errors in the experimental measurements and the numerical errors. The effect of carbon (C) on the ${M}_{S}^{{\rm{\varepsilon }}}$ temperature is well predicted by the assumption that the C-C interaction is insignificant in most csompositions. In addition, the structural stability considering the effect of the interstitial alloying element, C, was not significantly different from that of the pure Fe alloy, or from that after the addition of other substitute alloying elements.

A detailed analysis of the data can provide insight into measurement errors. As can be seen in Fig. 4b, there is a closer linear correlation for the alloy containing C, compared to the C-free alloys. It is believed that the initial austenite grain size difference is large and the ${M}_{S}^{{\rm{\varepsilon }}}$ temperature error is also large in C-free steel, because the C-free steel has higher grain growth rate of austenite^38^. Yang and Bhadeshia proposed a model that shows the influence of the parent austenite grain size on the *M*_*s*_ temperature and compared it with experimental measurements^39^. The ${M}_{S}^{{\rm{\varepsilon }}}$ temperature is also affected by the austenite grain size, similar to *α*′-martensite, and shows a difference at about 30 K with grain size differences of 10 *μm* and 200 *μm*. The relationship derived here reflects the effect of alloying elements alone, but has limitations in that it cannot reflect data scattering regarding the difference in the austenite grain size. In order to reduce the scattering of the ${M}_{S}^{{\rm{\varepsilon }}}$ temperature for the same compositions, it is necessary to modify the ${M}_{S}^{{\rm{\varepsilon }}}$ temperature reflecting the austenite grain size effect. In the case of the Fe-0.0017C-24.03 Mn wt% alloy, Δ*H* ^*γε*^(0 K) was calculated to be −0.505 kJ mol^−1^ and the ${M}_{S}^{{\rm{\varepsilon }}}$ temperature measurement result showed a 54 K difference (421 K^40^ and 367 K^41^) depending on the measurement method. Given the error, it can be seen that there is a fairly precise linear relationship for C containing alloys.

Figure 4b shows the Fe-Mn binary system, and the higher the amount of Mn, the lower the obtained ${M}_{S}^{{\rm{\varepsilon }}}$ temperature was. The data for approximately the same composition, 25.0 to 25.5 at% Mn, showed a large scatter of measurements (291–390 K), and this is corresponding to the magnetic ordering of austenite. If the parent austenite is in a paramagnetic state assuming completely disordered localized moments, then the magnetic entropy contribution *S*_*mag*_(*μ*_*i*_) for the magnetic moment *μ*_*i*_ of atom *i* can be estimated *S*_*mag*_(*μ*_*i*_) = *k*_*B*_log(*μ*_*i*_ + 1) with Boltzmann’s constant *k*_*B*_^42^. Below *T*_*N*_(*γ*), this magnetic entropy contribution is reduced by anti-ferromagnetic ordering, making the parent austenite more stable. The *T*_*N*_(*γ*) of austenite is raised by the addition of Mn, and this has an enhanced effect on suppressing the *ε*-martensitic transformation if the ${M}_{S}^{{\rm{\varepsilon }}}$ temperature is below *T*_*N*_(*γ*)^34^. Thus, in the *ε*-martensitic transformation, there is non-linear behavior around *T*_*N*_(*γ*), where the slope of the ${M}_{S}^{{\rm{\varepsilon }}}$ temperature changes depending on the amount of Mn. In addition, overlapping of the magnetic transition and structure transformation makes it difficult to detect the ${M}_{S}^{{\rm{\varepsilon }}}$ temperature, which causes large data scattering. This is an essential factor underlying the prediction error.

### Effect of alloying elements on ${M}_{S}^{\varepsilon }$ temperature

**Figure 5:**
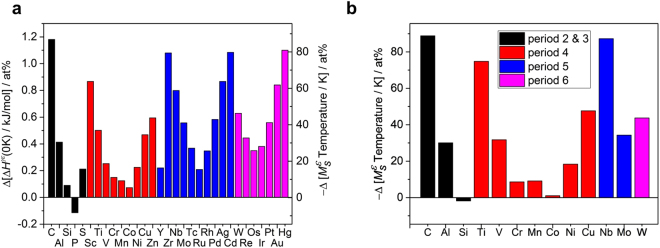
Alloying element effects on ${M}_{S}^{{\rm{\varepsilon }}}$ temperature. (**a)** Relative change of the enthalpy difference Δ*H* ^*γε*^(0 K) by 1 at% elements addition as compared to the Fe-20Mn at% and corresponding to ${M}_{S}^{{\rm{\varepsilon }}}$ temperature changes (right axis) according to the linear relationship of this study. (**b)** The regression coefficients that represent the contribution of each element to the prediction of the ${M}_{S}^{{\rm{\varepsilon }}}$ temperature according to the multiple linear regression model. Each value in Fig. 1b was rearranged to a value corresponding to 1 at%.

Based on the linear relationship of Δ*H* ^*γε*^(0 K) and the ${M}_{S}^{{\rm{\varepsilon }}}$ temperature, the effect of various elements on ${M}_{S}^{{\rm{\varepsilon }}}$ temperature was obtained. Figure 5a shows the change in lattice stability Δ*H* ^*γε*^(0 K) per 1 at% addition of element X in the Fe-20Mn-X at% system. The ${M}_{S}^{{\rm{\varepsilon }}}$ temperature was estimated to be as 397.5 K by Eq. (3) with Δ*H* ^*γε*^(0 K) = −1.085 kJ mol^−1^ for Fe-20Mn at%. The change of Δ*H* ^*γε*^(0 K) per 1 at% of element X was investigated for the interstitial element C; for the elements Al, Si, P, and S belonging to 3rd period; for the elements Sc, Ti, V, Cr, Mn, Co, Ni, Cu, and Zn belonging to 4th period; for the elements Y, Zr, Nb, Mo, Tc, Ru, Rh, Pd, Ag, and Cd belonging to 5th period; and for the elements W, Re, Os, Ir, Pt, Au, and Hg belonging to 6th period. The change in ${M}_{S}^{{\rm{\varepsilon }}}$ temperature as determined by Eq. (3) is plotted on the right axis. Figure 5b shows the effect of each element predicted through multiple linear regression, which is summarized in Fig. 1b, according to the atomic number with a factor corresponding to at% instead of wt%. The same ${M}_{S}^{{\rm{\varepsilon }}}$ temperature change scale is shown for comparison with Fig. 5a.

The Δ*H* ^*γε*^(0 K) increased by 1.181, 0.413, 0.149, 0.125, 0.073, 0.225, and 0.468 kJ mol^−1^ per 1 at% added of C, Al, Cr, Mn, Co, Ni, and Cu, respectively. It is predicted that this will lower the ${M}_{S}^{{\rm{\varepsilon }}}$ temperature by 88.0, 30.4, 11.0, 9.3, 5.4, 16.6, and 34.4 K, respectively. This is in agreement with the coefficients 88.0, 30.1, 8.7, 9.1, 1.1, 18.4, and 47.6 K for each element determined by multiple linear regression. In the case of Si addition, the Δ*H* ^*γε*^(0 K) was estimated to be increased by 0.090 kJ mol^−1^ and have a correspondingly lower ${M}_{S}^{{\rm{\varepsilon }}}$ temperature by 6.6 K, while the coefficient of the multiple linear regression was negative. As shown above, the multiple linear regression model does not reflect non-linearity, while the Si effect increases Δ*H* ^*γε*^(0 K) from 0 to 7 at%, and decreases it in the region above 7 at%. It is expected that the addition of Ti, V, Nb, Mo, and W at 1 at% will increase Δ*H* ^*γε*^(0 K) to of 0.501, 0.253, 0.799, 0.558, and 0.628 kJ mol^−1^, respectively, and lower the ${M}_{S}^{{\rm{\varepsilon }}}$ temperature to 36.9, 18.6, 58.8, 41.0, and 46.2 K, respectively. This is predicted to be small overall compared with the coefficients of multiple linear regression models 74.9, 31.7, 87.3, 34.3, and 43.8 K, but it shows qualitatively the same trend as Δ*H* ^*γε*^(0 K). The large error in the Ti, V, Nb, Mo, and W coefficients is probably due to insufficient number of data used to predict the coefficients. In addition, the influence due to other independent elements such as Mn is not reflected.

We can also deduce the effect of other elements that have not been subjected the actual experiment. For example, addition of 1 at% P will lower Δ*H* ^*γε*^(0 K), which will increase the ${M}_{S}^{{\rm{\varepsilon }}}$ temperature. As all the elements used in this search except P were added, its addition is expected to increase Δ*H* ^*γε*^(0 K) and thereby lower the ${M}_{S}^{{\rm{\varepsilon }}}$ temperature. These effects show a certain regularity according to the atomic number. Elements in the Group VIII (Fe, Ru, Os) showed the lowest effect on the lattice stability, and the wider the distance from Group VIII, the greater the effect on the lattice stability per 1 at%. This shows that the FCC and HCP lattice stability tended to be similar to that predicted by Pettifor’s theory^43^, which is determined by the electron occupancy of the *d*-orbital. Therefore, the effect on the ${M}_{S}^{{\rm{\varepsilon }}}$ temperature is mainly dominated by the occupancy of the *d*-orbitals.

In order to confirm the effect of alloying elements in the proposed model, two samples having compositions with different P content were prepared and ${M}_{S}^{{\rm{\varepsilon }}}$ temperature was measured. A sample was prepared by adding a certain amount of P based on Fe-Mn binary. P is an alloying element whose influence on ${M}_{S}^{{\rm{\varepsilon }}}$ temperature has not been clarified by previous experiments. The chemical compositions of the prepared samples were Fe-20.8Mn at% and Fe-20.7Mn-0.27P at%, respectively. For the Fe-20.8Mn at% sample, the measured ${M}_{S}^{{\rm{\varepsilon }}}$ temperature, 387 K, is about 3 K lower than the value of 390 K predicted using Eq. (3). The measured ${M}_{S}^{{\rm{\varepsilon }}}$ temperature for the Fe-20.7Mn-0.27P at% sample is 391 K, which is consistent with the result that the *ε*-martensitic transformation is enhanced by adding P. Also, the effect of ${M}_{S}^{{\rm{\varepsilon }}}$ temperature increment of 8 K per 1 at% is expected, which corresponds to 2.2 K per 0.27 at%. The predicted effect of P appears to be little underestimated compared to a 4 K temperature increase with experimental measurements. This is thought to be caused by the difference in Mn content. Experimental verification confirmed the reliability of the ${M}_{S}^{{\rm{\varepsilon }}}$ temperature prediction model based on the quantum-mechanical calculations.

### Driving force evaluation for *ε*-martensitic transformation

The original formulation of density functional theory was for the T = 0 K ground state^44^. Although it can be shown that the concept can be extended to finite electronic temperatures, historically, most calculations were restricted to zero temperature properties due to computational costs^45^. The molar Gibbs free energy of the crystal at a given temperature T and pressure P can be approximately decomposed into several contributing terms^45–48^.

5 $$G({\rm{T}},{\rm{P}})={E}_{tot}+{F}_{vib}+{F}_{conf}+{F}_{el}+{F}_{mag}+{\rm{PV}}.$$

The leading term *E*_*tot*_ is the total energy which is directly obtained from the electronic structure calculation allowed to be independent of temperatures. The term *F*_*vib*_, *F*_*conf*_, *F*_*el*_, and *F*_*mag*_ account for the vibrational, configurational, electronic, and magnetic contributions, respectively. The last one is the pressure-volume dependent term. The free energy change Δ*G* ^*γε*^(*T*) accompanying the *ε*-martensitic transformation involves Δ*H* ^*γε*^(0 K), and can be described as follows.

6 $${\rm{\Delta }}{G}^{{\rm{\gamma }}{\rm{\varepsilon }}}({\rm{T}})={\rm{\Delta }}{H}^{{\rm{\gamma }}{\rm{\varepsilon }}}\mathrm{(0}\,{\rm{K}})+{\rm{\Delta }}{H}^{ext}-{\rm{T}}{\rm{\Delta }}S$$

where Δ*H* ^*ext*^ includes all temperature independent contributions to free energy except for electronic total energy. This includes differences due to atomic volume differences, magnetic transitions, and errors due to the calculation approximations and parameters commented above. Here, Δ*S* is an entropy term including electronic, magnetic, vibrational, and configuration excitations.

A linear relationship between ${M}_{S}^{{\rm{\varepsilon }}}$ temperature and Δ*H* ^*γε*^(0 K) appears in the free energy configuration of Eq. (6). Assuming Δ*H* ^*ext*^ is independent of T, the free energy change at ${M}_{S}^{{\rm{\varepsilon }}}$ temperature is expressed as follows,

7 $${\rm{\Delta }}{G}^{\gamma {\rm{\varepsilon }}}({M}_{S}^{\varepsilon })={\rm{\Delta }}{H}^{\gamma {\rm{\varepsilon }}}\mathrm{(0}\,{\rm{K}})+{\rm{\Delta }}{H}^{ext}-{M}_{S}^{{\rm{\varepsilon }}}{\rm{\Delta }}S.$$

The driving force of the *ε*-martensitic transformation, ${\rm{\Delta }}{G}^{\gamma {\rm{\varepsilon }}}({M}_{S}^{{\rm{\varepsilon }}})$, varies slightly depending on the alloy composition, but the difference is less than 0.1 kJ mol^−1^ ^12,49,50^. The linear relationship between Δ*H* ^*γε*^(0 K) and ${M}_{S}^{{\rm{\varepsilon }}}$ temperature shows that Δ*H* ^*ext*^ and Δ*S* in Eq. (7) can be approximated as a constant due to the small degree of variation depending on the alloy composition. In the case of the Δ*S* value, it can be approximated as −0.0136 kJ mol^−1^ K from the comparison of Eqs (4) and (7). The Δ*H* ^*ext*^ value can be determined from the *T*_0_ temperature, which corresponds to Δ*G* ^*γε*^(*T*_0_) = 0 in the Fe-Mn binary system. For the Fe-28.45Mn at% system, Δ*H* ^*γε*^(0 K) = −0.025 kJ mol^−1^ was calculated and Δ*H* ^*ext*^ = −4.671 kJ mol^−1^ was determined from the experimentally measured result with *T*_0_ = 345.36 K in the system^49^.

**Figure 6:**
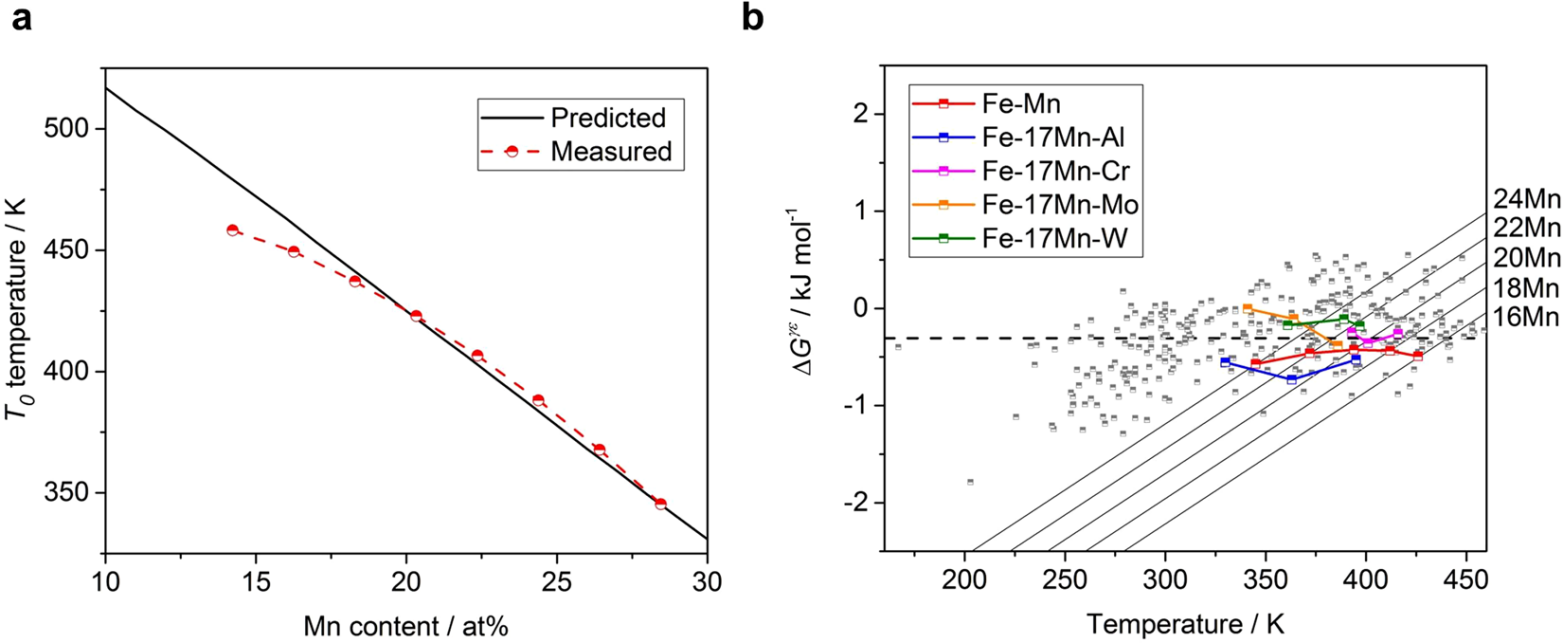
Prediction results according to Eq. (7) in the Fe-Mn binary and Fe-17Mn-X ternary system: (**a)**, Predicted and measured *T*_0_ temperature^49^ as a function of Mn content in the Fe-Mn binary system. (**b)** The change of free energy as a function of temperature with different Mn amount (at%) according to Eq. (7) in the Fe-Mn binary system, and calculated free energy change ${\rm{\Delta }}{G}^{\gamma {\rm{\varepsilon }}}({M}_{S}^{{\rm{\varepsilon }}})$ in the Fe-Mn^49^ (half-red square), Fe-17Mn-Al^63^ (half-blue square), Fe-17Mn-Cr^63^ (half-purple square), Fe-17Mn-Mo^63^ (half-yellow square) and Fe-17Mo-W^63^ (half-green square) systems for different amount of alloying addition (at%). The half-grey squares are the free energy change corresponding to the collected data. The half-red squares are the measured temperature and the corresponding free energy point at a given Mn composition. The black dashed line shows the average driving force for *ε*-martensitic transformation predicted in this study (−0.281 kJ mol^−1^).

The *T*_0_ temperatures and free energy changes during martensitic transformation in the Fe-Mn binary system are evaluated based on Eq. (7). The prediction of *T*_0_ temperatures is well correlated with the existing measurement data as shown in Fig. 6a. As mentioned above, the Fe-Mn system exhibits a non-linear tendency in which the slope of ${M}_{S}^{{\rm{\varepsilon }}}$ temperature as a function of the amount of Mn changes near *T*_*N*_(*γ*). The *T*_0_ temperature is mainly determined by the ${M}_{S}^{{\rm{\varepsilon }}}$ temperature, and there is also a non-linear tendency due to the magnetic ordering. Below 18 at% Mn, the mangetic entropy contribution increases, and the free energy model derived from linear relationship. Nevertheless, the error is within the range of data scattering by measurement within 30 K.

The free energy changes during martensitic transformation, ${\rm{\Delta }}{G}^{\gamma {\rm{\varepsilon }}}({M}_{S}^{{\rm{\varepsilon }}})$, show nearly normal distribution in Fig. 6b with mean of −0.281 kJ mol^−1^ and standard deviation of 0.39 kJ mol^−1^, unlike in thermodynamic calculations. This standard deviation corresponds to ±28.7 K at ${M}_{S}^{{\rm{\varepsilon }}}$ temperature. It is considered that the distribution of ${\rm{\Delta }}{G}^{\gamma {\rm{\varepsilon }}}({M}_{S}^{{\rm{\varepsilon }}})$ is reasonable when the measurement error considered. The average driving force, 281 J mol^−1^, is within the existing results of 68–120 J/mol^49^, 200–300 J/mol^50^, and 500–900 J/mol^51^. Figure 6b also shows the free energy changes at ${M}_{S}^{{\rm{\varepsilon }}}$ temperature in the ternary Fe-17Mn-Al, Fe-17Mn-Cr, Fe-17Mn-Mo, and Fe-17Mn-W in the unit of at%. The Fe-Mn binary and Fe-17Mn-Al systems show underestimated ${M}_{S}^{{\rm{\varepsilon }}}$ temperature with a lower ${\rm{\Delta }}{G}^{\gamma {\rm{\varepsilon }}}({M}_{S}^{{\rm{\varepsilon }}})$ value than the average driving force. In contrast, the values of the Fe-17Mn-Mo and Fe-17Mn-W systems are located above the average driving force line and show a higher measured ${M}_{S}^{{\rm{\varepsilon }}}$ temperature than the predicted value. However, the overall free energy change is close to the average driving force and the ${M}_{S}^{{\rm{\varepsilon }}}$ is within the prediction error. This method allows a temperature-dependent free energy model to be established based on the quantum-mechanical calculations.

## Conclusions

On the basis of the quantum-mechanical calculations, we discuss the relationship between the stability of FCC and HCP lattice structures and the ${M}_{S}^{{\rm{\varepsilon }}}$ temperature of various alloy systems. We first note that there is a close linear relationship between the measured ${M}_{S}^{{\rm{\varepsilon }}}$ temperature and the crystal structure stability of the anti-ferromagnetic FCC as well as the paramagnetic HCP at the 0 K. Thus, the effect of each element on the ${M}_{S}^{{\rm{\varepsilon }}}$ temperature could be determined. The effect of each element on ${M}_{S}^{{\rm{\varepsilon }}}$ temperature is related to the occupancy of electrons filling in the *d*-orbital. As the distance between the element and the Group VIII widens, it was found that the degree of lowering the ${M}_{S}^{{\rm{\varepsilon }}}$ temperature by the addition of the same amount of element, was increased. Based on the measured *T*_0_ and ${M}_{S}^{{\rm{\varepsilon }}}$ temperature, the force driving the transformation to *ε*-martensite was calculated to be −0.281 kJ mol^−1^ on average, which is within the predicted range.

Our study is the first to show the relationships between the experimentally measured data of various compositions of steels, and quantum-mechanical calculations. Applying the alloy composition and temperature dependency to the results based on the density-functional theory was difficult problem. The coherent-potential approximation method was used to simulate the effect of alloys; so that the effect of alloying elements on the ${M}_{S}^{{\rm{\varepsilon }}}$ temperature could be deduced. It is believed that the quantum-mechanical calculations can be used to predict the characteristics of steel alloys, which are typical structural materials, and can now be used to design new alloys by predicting their martensitic transformation start temperature.

## Methods

### Data

A search of the literature revealed a large number of data sources some 322 combinations on chemical composition and ${M}_{S}^{{\rm{\varepsilon }}}$ temperatures^52^. Included were data on Mn corresponding to 13–35.9 wt%, and on twelve other elements (C, Ni, Cr, Al, Si, Mo, Co, Cu, Nb, Ti, V and W). Of the total data, 232 do not contain carbon. In addition, 68 data are distributed from 0 to 0.08 wt% or less carbon content, 10 data from 0.09 to 0.16 wt% or less, and 12 data from 0.16 to 0.35 wt%. For Mn, there were 59 data for Fe-Mn binary alloys, with Mn proportion ranging from 13.6 to 29.3 wt%. In the case of Mo, Ti, Nb, V, and W, there were 4, 4, 3, 3, and 3 data, respectively, with a specified amount added; so it is necessary to consider that there is an insufficient number of data to effectively grasp the tendency. In contrast, in cases of C, Mn, Ni, Cr, Al, Si, Co and Cu, a relatively wide variety of alloy systems are included, thereby ensuring reliability in understanding the tendency.

### DFT calculations

The lattice stability of random alloys was calculated using the EMTO-CPA method^53^. In the present calculations, the one-electron equations were solved using the full charge density (FCD)^31^ method and frozen-core approximation, i.e., the core states were fixed to the initial atomic states. All self-consistent calculations were performed using the local density approximation (LDA) of the effective exchange-correlation potential, and the total energies were obtained using the Perdew-Burke-Ernzerhof (PBE) realization of the generalized gradient approximation (GGA)^54^. Poisson’s equation was solved within the spherical cell approximation(SCA)^53^. The Green’s function for the valence states was calculated for 16 complex energy points distributed exponentially on a semi-circular contour. The EMTO basis set included *s*, *p*, *d*, and *f* orbitals. We used more than 2000 inequivalent k-points in the irreducible wedge of the FCC, HCP, and BCT Brillouin zone, which ensured relative accuracy 1.0 × 10^−3^ eV/atom in the total energy differences. Self-consistency was assumed when the distances between the input and output total energies and fermi level were less than 1.3 × 10^−7^ eV and 1.3 × 10^−6^ eV, respectively. The convergence of these computational parameters was carefully checked.

Austenite with a FCC structure was simulated using a primitive unit cell containing one lattice site and a BCT structure including two lattice sites. We include different magnetic structures for austenite, including nonmagnetic (NM), paramagnetic (PM), and anti-ferromagnetic (AFM) state. For austenite, the AFM state is known to be stable at low temperature^55,56^. This was realized using a BCT unit cell for the structure in which the spin up-down state is crossed at every (001)-plane. According to the results of previous studies, the structure in which the spin direction changes every two layers was calculated to be the most stable, but the energy difference was found to be insignificant^57,58^. The *ε*-martensite, which has the HCP structure, was simulated assuming a para-magnetic state based on a primitive unit cell containing two lattice sites. The paramagnetic configuration of the FCC and HCP alloys was simulated by means of the disordered local moments (DLM) model^59^. The DLM model was shown to describe the random distribution of the local magnetic moments of the paramagnetic state of metals, above the magnetic transition temperature.

The crystal structures of different alloys and their magnetic states were optimized. For FCC, the energy for nine points was calculated in 0.5% increments from −2% to 2% based on the lattice parameter of *a*_*γ*_ = 3.6 Å, which is the experimental value of austenite. In the case of *ε*-martensite with HCP structure, seven *c*/*a* ratios (1.580, 1.585, 1.590, 1.595, 1.600, 1.605, and $\sqrt{\mathrm{8/3}}=1.632$, the ideal ratio for FCC structure) were included. The energy for nine points was calculated in 0.5% increments from −2% to 2% based on ${a}_{0}={a}_{\gamma }/\sqrt{2}=2.74$ Å which corresponds to the austenite lattice parameter. The optimum lattice parameter, atomic volume, and *c*/*a* ratio were determined based on calculations for different 63 combinations of *a*_0_ and *c*_0_. Equilibrium volume, equilibrium total energy, and bulk modulus were calculated using a third-order Birch-Murnaghan fit after calculating energy values per atomic volume in each structure^60^. The optimum *c*/*a* ratio was determined by fitting the equilibrium total energy function to the *c*/*a* ratio with a fourth order polynomial. The element concentrations of each alloy were converted to mole fractions and rounded to an even number at the third decimal place to achieve the same amount of alloying in NM, AFM, and DLM simulations. The lattice stability effect of carbon as an interstitial element, was applied using the results calculated based on the FCD-EMTO method in a previous study^14^. Based on the value of Δ*H* ^*γε*^(0 K) = 3.66 kJ/mol calculated for the 32Fe-1C structure, which corresponds to 0.667 wt% C, the result was 5.49 kJ/mol per 1 wt%. The carbon range of interest is less than 0.35 wt%, and the dilute solution linearly approximated the effect of C, assuming that the effect of C-C interaction is small. We neglected all thermal contributions in the present calculations. The datasets generated during the current study are available in Supplementary Table 1 and the ref.^61^.

### Measurements of ${M}_{S}^{\varepsilon }$ temperature

A set of alloys was prepared as 400 g melts, which were cast and vacuum sealed. They were then homogenized for two days at 1473 K, after which their chemical compositions were measured. Cylindrical samples with 3 mm diameter and 10 mm length were machined and studied using a dilatometer. The samples were heated at 2 K s^−1^ under a vacuum to 873 K for 1 min, and then quenched at 10 K s^−1^ using argon gas to room temperature. The offset method was used to determine the ${M}_{S}^{{\rm{\varepsilon }}}$ temperature, and the critical strain value was set at 2% of the strain amount at the completion of the transformation. More than two experiments were conducted on the same composition, and the measured values were distributed within 1 K.

## Electronic supplementary material

Table S1

## References

[1] Kaufman L, Cohen M (1958). Thermodynamics and kinetics of martensitic transformations. Progress in Metal Physics.

[2] Olson G, Cohen M (1976). A general mechanism of martensitic nucleation: Part i. general concepts and the fcc-hcp transformation. Metallurgical Transactions A.

[3] Bhadeshia H (1981). Driving force for martensitic transformation in steels. Metal Science.

[4] Kaufman, L., Radcliffe, S. & Cohen, M. Thermodynamics of the bainite reaction. *Decomposition of Austenite by Diffusional Processes* 313–352 (1962).

[5] Ghosh G, Olson G (1994). Kinetics of fcc-bcc heterogeneous martensitic nucleation:i. the critical driving force for athermal nucleation. Acta Metallurgica et Materialia.

[6] Ghosh G, Olson G (2001). Computational thermodynamics and the kinetics of martensitic transformation. Journal of Phase Equilibria.

[7] Frommeyer G, Brüx U, Neumann P (2003). Supra-ductile and high-strength manganese-trip/twip steels for high energy absorption purposes. ISIJ international.

[8] Lee T-H (2017). Self-twinning in solid-state decomposition. Acta Materialia.

[9] Mosecker L, Saeed-Akbari A (2013). Nitrogen in chromium–manganese stainless steels: a review on the evaluation of stacking fault energy by computational thermodynamics. Science and Technology of Advanced Materials.

[10] Adler P, Olson G, Owen W (1986). Strain hardening of hadfield manganese steel. Metallurgical and Materials Transactions A.

[11] Nakano J (2013). A thermo-mechanical correlation with driving forces for hcp martensite and twin formations in the fe–mn–c system exhibiting multicomposition sets. Science and Technology of Advanced Materials.

[12] Yang H-S, Jang J, Bhadeshia H, Suh D (2012). Critical assessment: Martensite-start temperature for the *γ*-*ε* transformation. Calphad.

[13] Pisarik S, Van Aken D (2016). Thermodynamic driving force of the *γ*-*ε* transformation and resulting ms temperature in high-mn steels. Metallurgical and Materials Transactions A.

[14] Lu S (2017). Stacking fault energy of c-alloyed steels: The effect of magnetism. Acta Materialia.

[15] Gebhardt T (2010). Ab initio lattice stability of fcc and hcp fe–mn random alloys. Journal of Physics: Condensed Matter.

[16] Gebhardt T (2011). The influence of additions of al and si on the lattice stability of fcc and hcp fe–mn random alloys. Journal of Physics: Condensed Matter.

[17] Stripp KF, Kirkwood JG (1954). Lattice vibrational spectrum of imperfect crystals. The Journal of Chemical Physics.

[18] Wojtowicz PJ, Kirkwood JG (1960). Contribution of lattice vibrations to the order-disorder transformation in alloys. The Journal of Chemical Physics.

[19] Bellaiche L, Garca A, Vanderbilt D (2000). Finite-temperature properties of pb (zr 1- x ti x) o 3 alloys from first principles. Physical Review Letters.

[20] Sanchez JM, Ducastelle F, Gratias D (1984). Generalized cluster description of multicomponent systems. Physica A: Statistical Mechanics and its Applications.

[21] Zunger A, Wei S-H, Ferreira L, Bernard JE (1990). Special quasirandom structures. Physical Review Letters.

[22] Soven P (1967). Coherent-potential model of substitutional disordered alloys. Physical Review.

[23] Taylor D, Vashishta P (1972). Electron-phonon interaction and superconductivity in in-tl alloys. Physical Review B.

[24] Gyorffy B (1972). Coherent-potential approximation for a nonoverlapping-muffin-tin-potential model of random substitutional alloys. Physical Review B.

[25] Gunnarsson O, Jepsen O, Andersen O (1983). Self-consistent impurity calculations in the atomic-spheres approximation. Physical Review B.

[26] Abrikosov I, Skriver HL (1993). Self-consistent linear-muffin-tin-orbitals coherent-potential technique for bulk and surface calculations: Cu-ni, ag-pd, and au-pt random alloys. Physical Review B.

[27] Vitos L, Nilsson J-O, Johansson B (2006). Alloying effects on the stacking fault energy in austenitic stainless steels from first-principles theory. Acta Materialia.

[28] Vitos L, Korzhavyi PA, Johansson B (2006). Evidence of large magnetostructural effects in austenitic stainless steels. Physical review letters.

[29] Vitos L, Korzhavyi PA, Johansson B (2002). Modeling of alloy steels. Materials Today.

[30] Vitos L, Abrikosov I, Johansson B (2001). Anisotropic lattice distortions in random alloys from first-principles theory. Physical review letters.

[31] Vitos L (2001). Total-energy method based on the exact muffin-tin orbitals theory. Physical Review B.

[32] Sundman B, Jansson B, Andersson J-O (1985). The thermo-calc databank system. Calphad.

[33] Endoh Y, Ishikawa Y (1971). Antiferromagnetism of *γ* iron manganes alloys. Journal of the Physical Society of Japan.

[34] Palumbo M (2008). Thermodynamics of martensitic transformations in the framework of the calphad approach. Calphad.

[35] Acet M, Schneider T, Gehrmann B, Wassermann E (1995). The magnetic aspects of the *γ*-*α* and *γ*-*ε* martensitic transformations in fe-mn alloys. Le Journal de Physique IV.

[36] Jin J-E, Jung M, Lee C-Y, Jeong J, Lee Y-K (2012). Néel temperature of high mn austenitic steels. Metals and Materials International.

[37] Dumay A, Chateau J-P, Allain S, Migot S, Bouaziz O (2008). Influence of addition elements on the stacking-fault energy and mechanical properties of an austenitic fe–mn–c steel. Materials Science and Engineering: A.

[38] Akbari G, Sellars C, Whiteman J (1995). Austenite and ferrite grain sizes in interstitial free steel. Materials science and technology.

[39] Yang H-S, Bhadeshia H (2009). Austenite grain size and the martensite-start temperature. Scripta materialia.

[40] Tomota Y, Morioka Y, Nakagawara W (1998). Epsilon martensite to austenite reversion and related phenomena in fe? 24mn and fe? 24mn? 6si alloys. Acta materialia.

[41] Tomota Y, Nakagawara W, Tsuzaki K, Maki T (1992). Reversion of stress-induced martensite and two-way shape memory in fe-24mn and fe-24mn-6si alloys. Scripta metallurgica et materialia.

[42] Grimvall G (1989). Spin disorder in paramagnetic fcc iron. Physical Review B.

[43] Pettifor D (2003). Electron theory in materials modeling. Acta materialia.

[44] Hohenberg P, Kohn W (1964). Inhomogeneous electron gas. Physical review.

[45] Neugebauer J, Hickel T (2013). Density functional theory in materials science. Wiley Interdisciplinary Reviews: Computational Molecular Science.

[46] Rogal, J. & Reuter, K. Ab initio atomistic thermodynamics for surfaces: A primer. Tech. Rep., DTIC Document (2006).

[47] Reuter K, Scheffler M (2001). Composition, structure, and stability of ruo 2 (110) as a function of oxygen pressure. Physical Review B.

[48] Moruzzi V, Janak J, Schwarz K (1988). Calculated thermal properties of metals. Physical Review B.

[49] Lee Y-K, Choi C (2000). Driving force for *γ*-*ε* martensitic transformation and stacking fault energy of *γ* in fe-mn binary system. Metallurgical and Materials Transactions A.

[50] Ishida K (1977). Effect of alloying elements on the critical driving force of martensitic transformation in iron alloys. Scripta Metallurgica.

[51] Holden A, Bolton J, Petty E (1971). Structure and properties of fe-mn alloys. J Iron Steel Inst.

[52] Yang, H. Data for epsilon martensite-start temperatures in steels URL http://cml.postech.ac.kr/2011/epsilon.zip (2011).

[53] Vitos, L. *Computational quantum mechanics for materials engineers: the EMTO method and applications* (Springer Science & Business Media, 2007).

[54] Perdew JP, Burke K, Ernzerhof M (1996). Generalized gradient approximation made simple. Physical review letters.

[55] Bisanti P, Mazzone G, Sacchetti F (1987). Electronic structure of fcc fe-mn alloys. ii. spin-density measurements. Journal of Physics F: Metal Physics.

[56] Schulthess T, Butler W, Stocks G, Maat S, Mankey G (1999). Noncollinear magnetism in substitutionally disordered face-centered-cubic femn. Journal of applied physics.

[57] Stocks GM (2002). On the magnetic structure of *γ*-femn alloys. Journal of applied physics.

[58] Jiang D, Carter EA (2003). Carbon dissolution and diffusion in ferrite and austenite from first principles. Physical Review B.

[59] Staunton J, Gyorffy B, Pindor A, Stocks G, Winter H (1984). The disordered local momen picture of itinerant magnetism at finite temperatures. Journal of magnetism and magnetic materials.

[60] Birch F (1952). Elasticity and constitution of the earth’s interior. Journal of Geophysical Research.

[61] Jang, J. H. Quantum-mechanical calculations for epsilon martensite-start temperatures in steels URL https://github.com/impurity80/script/blob/master/emstemperature.xlsx (2017). 10.1038/s41598-017-18230-z PMC5736593 29259306

[62] Umebayashi H, Ishikawa Y (1966). Antiferromagnetism of *γ* fe-mn alloys. Journal of the Physical Society of Japan.

[63] Ishida K, Nishizawa T (1974). Effect of alloying elements on stability of epsilon iron. Transactions of the Japan Institute of Metals.

