# Supplementary material for: Quantum-mechanical analysis of effect of alloying elements on ε-martensite start temperature of steels
Source: Sci Rep. 2017 Dec 19;7:17860. doi: 10.1038/s41598-017-18230-z (PMC5736593; doi:10.1038/s41598-017-18230-z)
Supplement: Supplementary file 1 — Table S1 [file 41598_2017_18230_MOESM1_ESM.pdf]

Quantum-mechanical analysis of effect of alloying elements on ε-martensite start temperature of steels

J. H. Jang[1,*], J. Moon[1], H.-Y. Ha[1], T.-H. Lee[1], D.-W. Suh[2]

[1] Ferrous Alloy Department, Korea Institute of Materials Science, Republic of Korea

[2] Graduate Institute of Ferrous Technology, POSTECH, Republic of Korea

Table S1. The M_S^{ε} temperature data compiled from the literature and calculated total energies for FCC and HCP structure, respectively. Compositions are in wt%.

Alloy	C	Mn	Ni	Cr	Al	Si	Mo	Co	Cu	Nb	Ti	V	W	Ms	energy (HCP) / eV	energy (FCC) / eV	delta (eV)	delta (kJ/mol)	delta (kJ/mol) wit C
1	0	31.5	0	0	0	6	0	0	0	0	0	0	0	293	-491.46	-491.46	0.0030	0.288	0.288
2	0	27.6	0	5	0	6.1	0	0	0	0	0	0	0	283	-481.51	-481.51	0.0045	0.429	0.429
3	0	20.4	5	8	0	5	0	0	0	0	0	0	0	261	-503.48	-503.49	0.0044	0.425	0.425
4	0	19.9	5	10.1	0	5	0	0	0	0	0	0	0	226	-497.92	-497.93	0.0050	0.483	0.483
5	0	17.5	5.5	8.5	0	5.1	0	0	0	0	0	0	0	254	-507.15	-507.15	0.0023	0.223	0.223
6	0	16.9	6.3	10	0	5	0	0	0	0	0	0	0	254	-506.09	-506.10	0.0032	0.310	0.310
7	0	16.5	5	8.4	0	5.1	0	0	0	0	0	0	0	245	-507.95	-507.95	0.0011	0.103	0.103
8	0	16	4.9	11.6	0	5	0	0	0	0	0	0	0	267	-499.61	-499.61	0.0030	0.286	0.286
9	0	13.7	4.4	8	0	5	0	0	0	0	0	0	0	275	-513.81	-513.81	-0.0020	-0.193	-0.193
10	0	13.3	5.8	9.6	0	4.9	0	0	0	0	0	0	0	277	-513.36	-513.36	0.0000	-0.003	-0.003
11	0	13	6.8	11.4	0	4.7	0	0	0	0	0	0	0	244	-511.96	-511.96	0.0016	0.151	0.151
12	0	25	0	7	0	6	0	0	0	0	0	0	0	279	-480.46	-480.46	0.0021	0.206	0.206
13	0	21	0	9	0	6	0	0	0	0	0	0	0	285	-480.89	-480.89	-0.0035	-0.336	-0.336
14	0.07	20.3	0	2.14	0	0	0	0	0	0	0	0	0	383	-556.02	-556.02	-0.0067	-0.644	-0.259
15	0.11	16.8	0	0.18	1.45	0	0	0	0	0	0	0	0	287	-552.74	-552.74	-0.0025	-0.238	0.367
16	0.35	18.1	0	0.19	0.18	0	0	0	0	0	0	0	0	263	-564.71	-564.70	-0.0117	-1.127	0.796
17	0	30.3	0	0	0	6.06	0	0	0	0	0	0	0	323	-492.24	-492.24	0.0017	0.166	0.166
18	0	26.4	0	0	0	5.87	0	0	0	0	0	0	0	354	-500.91	-500.90	-0.0025	-0.244	-0.244
19	0	23.4	0	0	0	5.91	0	0	0	0	0	0	0	372	-505.84	-505.84	-0.0059	-0.562	-0.562
20	0.04	20.2	0	4.1	0	0	0	0	0	0	0	0	0	373	-549.48	-549.48	-0.0034	-0.331	-0.111
21	0.16	19.7	0	0	0	0	0	0	0	0	0	0	0	322	-564.33	-564.32	-0.0115	-1.102	-0.223
22	0	24	0	0	0	6	0	0	0	0	0	0	0	378	-503.81	-503.80	-0.0052	-0.502	-0.502
23	0.02	17.7	0	0	0	0	0	0	0	0	0	0	0	428	-567.50	-567.49	-0.0139	-1.333	-1.223
24	0.1	17.8	0	0	0	0	0	0	0	0	0	0	0	373	-567.50	-567.49	-0.0139	-1.333	-0.783
25	0.2	17.9	0	0	0	0	0	0	0	0	0	0	0	328	-567.50	-567.49	-0.0139	-1.333	-0.234
26	0.28	17.3	0	0	0	0	0	0	0	0	0	0	0	302	-568.56	-568.55	-0.0147	-1.410	0.128
27	0	24.3	0	0	0	3	0	0	0	0	0	0	0	396	-528.86	-528.85	-0.0030	-0.290	-0.290
28	0	24.9	0	0	0	2.2	0	0	0	0	0	0	0	388	-535.29	-535.28	-0.0023	-0.221	-0.221
29	0	26	0	0	0	3.7	0	0	0	0	0	0	0	379	-520.16	-520.15	-0.0011	-0.104	-0.104
30	0	27.3	0	0	0	3.4	0	0	0	0	0	0	0	376	-519.65	-519.65	0.0007	0.066	0.066
31	0	27.9	0	0	0	2.6	0	0	0	0	0	0	0	362	-526.07	-526.08	0.0017	0.160	0.160
32	0	27.8	0	0	0	3.2	0	0	0	0	0	0	0	375	-520.90	-520.90	0.0012	0.114	0.114
33	0	27.6	0	0	0	3.6	0	0	0	0	0	0	0	335	-518.31	-518.31	0.0009	0.087	0.087
34	0	28.9	0	0	0	4	0	0	0	0	0	0	0	361	-512.28	-512.28	0.0022	0.211	0.211
35	0	31.5	0	0	0	4.4	0	0	0	0	0	0	0	298	-504.12	-504.13	0.0043	0.414	0.414
36	0	32	0	0	0	3.9	0	0	0	0	0	0	0	300	-507.96	-507.97	0.0058	0.559	0.559
37	0	32.9	0	0	0	3.5	0	0	0	0	0	0	0	295	-510.12	-510.13	0.0071	0.684	0.684
38	0	33.9	0	0	0	4.1	0	0	0	0	0	0	0	281	-502.83	-502.84	0.0078	0.750	0.750
39	0	35.9	0	0	0	4	0	0	0	0	0	0	0	263	-500.28	-500.29	0.0101	0.972	0.972
40	0.02	29.4	0	0	0	6	0	0	0	0	0	0	0	285	-494.99	-494.99	0.0004	0.036	0.146
41	0.01	28.3	0	0	0	6.8	0	0	0	0	0	0	0	323	-490.24	-490.24	-0.0014	-0.136	-0.064
42	0.02	29	0	5	0	6.9	0	0	0	0	0	0	0	283	-472.53	-472.54	0.0078	0.748	0.847
43	0	26.8	0	0	0	0	0	0	0	0	0	0	0	316	-551.63	-551.63	-0.0019	-0.182	-0.182
44	0	26.3	0	0	0	2.11	0	0	0	0	0	0	0	335	-533.80	-533.80	-0.0006	-0.055	-0.055
45	0	26.6	0	0	0	4.06	0	0	0	0	0	0	0	350	-515.53	-515.53	-0.0001	-0.009	-0.009
46	0	26.7	0	0	0	7.12	0	0	0	0	0	0	0	300	-490.12	-490.12	-0.0035	-0.335	-0.335
47	0	16	0	0	0	0	0	0	0	0	0	0	0	426	-570.68	-570.66	-0.0164	-1.571	-1.571

48	0	18	0	0	0	0	0	0	0	0	0	0	0	412	-567.15	-567.14	-0.0136	-1.306	-1.306
49	0	20	0	0	0	0	0	0	0	0	0	0	0	394	-563.62	-563.61	-0.0110	-1.056	-1.056
50	0	22	0	0	0	0	0	0	0	0	0	0	0	372	-560.10	-560.09	-0.0083	-0.796	-0.796
51	0	24	0	0	0	0	0	0	0	0	0	0	0	345	-556.22	-556.21	-0.0054	-0.514	-0.514
52	0.01	15.7	0	0	0	0.01	0	0	0	0	0	0	0	437	-571.03	-571.02	-0.0166	-1.597	-1.564
53	0	16.4	0	0	0	0.02	0	0	0	0	0	0	0	428	-569.97	-569.96	-0.0158	-1.513	-1.497
54	0	19.8	0	0	0	0.01	0	0	0	0	0	0	0	394	-563.98	-563.97	-0.0112	-1.076	-1.070
55	0	24.4	0	0	0	0.02	0	0	0	0	0	0	0	348	-555.86	-555.86	-0.0051	-0.488	-0.477
56	0	17.7	0	0	0	0	0	0	0	0	0	0	0	397	-567.50	-567.49	-0.0139	-1.333	-1.333
57	0	17.7	0	0	0	0	0	0	0	0	0	0	0	414	-567.50	-567.49	-0.0139	-1.333	-1.333
58	0	17.7	0	0	0	0	0	0	0	0	0	0	0	419	-567.50	-567.49	-0.0139	-1.333	-1.333
59	0	17.7	0	0	0	0	0	0	0	0	0	0	0	423	-567.50	-567.49	-0.0139	-1.333	-1.333
60	0	17.7	0	0	0	0	0	0	0	0	0	0	0	425	-567.50	-567.49	-0.0139	-1.333	-1.333
61	0	24	0	0	0	0	0	0	0	0	0	0	0	421	-556.22	-556.21	-0.0054	-0.514	-0.505
62	0	24.3	0	0	0	5.88	0	0	0	0	0	0	0	392	-504.43	-504.43	-0.0049	-0.471	-0.456
63	0.02	17.1	0	0	0	0	0	0	0	0	0	0	0	426	-568.56	-568.55	-0.0147	-1.410	-1.300
64	0.03	13.4	0	0	0	0	0	0	0	0	0	0	0	454	-575.27	-575.25	-0.0197	-1.896	-1.759
65	0.03	16.2	0	0	0	0	0	0	0	0	0	0	0	424	-570.33	-570.31	-0.0161	-1.546	-1.392
66	0.02	17	0	0	0	0	0	0	0	0	0	0	0	415	-568.92	-568.90	-0.0150	-1.436	-1.332
67	0.02	21.3	0	0	0	0	0	0	0	0	0	0	0	375	-561.15	-561.15	-0.0091	-0.873	-0.758
68	0.02	23	0	0	0	0	0	0	0	0	0	0	0	355	-558.33	-558.33	-0.0070	-0.669	-0.571
69	0.02	23.2	0	0	0	0	0	0	0	0	0	0	0	387	-557.98	-557.97	-0.0067	-0.644	-0.561
70	0.01	23.3	0	0	0	0	0	1.23	0	0	0	0	0	384	-560.24	-560.23	-0.0058	-0.552	-0.492
71	0.02	23.2	0	0	0	0	0	2.3	0	0	0	0	0	383	-562.41	-562.41	-0.0053	-0.512	-0.413
72	0.01	23.1	0	0	0	0	0	3.42	0	0	0	0	0	386	-564.94	-564.93	-0.0052	-0.497	-0.437
73	0.02	17.2	0	0	0	0	0	0	0	0	0	0	0	421	-568.56	-568.55	-0.0147	-1.410	-1.328
74	0.02	17.2	0	0	0	0	0	0	0	0	0	0	0	425	-568.56	-568.55	-0.0147	-1.410	-1.328
75	0.01	20.2	0	0	0	0	0	0	0	0	0	0	0	410	-563.27	-563.26	-0.0107	-1.031	-0.976
76	0.02	23.3	0	0	0	0	0	0	0	0	0	0	0	392	-557.63	-557.62	-0.0064	-0.613	-0.530
77	0	13.7	0	0	0	0	0	0	0	0	0	0	0	422	-574.91	-574.89	-0.0195	-1.871	-1.871
78	0	13.7	0	0	0	0	0	0	0	0	0	0	0	416	-574.91	-574.89	-0.0195	-1.871	-1.871
79	0	15.9	0	0	0	0	0	0	0	0	0	0	0	432	-570.68	-570.66	-0.0164	-1.571	-1.571
80	0	15.9	0	0	0	0	0	0	0	0	0	0	0	426	-570.68	-570.66	-0.0164	-1.571	-1.571
81	0	15.9	0	0	0	0	0	0	0	0	0	0	0	444	-570.68	-570.66	-0.0164	-1.571	-1.571
82	0	15.9	0	0	0	0	0	0	0	0	0	0	0	440	-570.68	-570.66	-0.0164	-1.571	-1.571
83	0	19.3	0	0	0	0	0	0	0	0	0	0	0	401	-564.68	-564.67	-0.0118	-1.131	-1.131
84	0	19.3	0	0	0	0	0	0	0	0	0	0	0	395	-564.68	-564.67	-0.0118	-1.131	-1.131
85	0	19.3	0	0	0	0	0	0	0	0	0	0	0	418	-564.68	-564.67	-0.0118	-1.131	-1.131
86	0	21.9	0	0	0	0	0	0	0	0	0	0	0	396	-560.10	-560.09	-0.0083	-0.796	-0.796
87	0	21.9	0	0	0	0	0	0	0	0	0	0	0	404	-560.10	-560.09	-0.0083	-0.796	-0.796
88	0	21.9	0	0	0	0	0	0	0	0	0	0	0	376	-560.10	-560.09	-0.0083	-0.796	-0.796
89	0	24.7	0	0	0	0	0	0	0	0	0	0	0	380	-555.16	-555.15	-0.0045	-0.436	-0.436
90	0	24.7	0	0	0	0	0	0	0	0	0	0	0	376	-555.16	-555.15	-0.0045	-0.436	-0.436
91	0	24.7	0	0	0	0	0	0	0	0	0	0	0	372	-555.16	-555.15	-0.0045	-0.436	-0.436
92	0	24.7	0	0	0	0	0	0	0	0	0	0	0	365	-555.16	-555.15	-0.0045	-0.436	-0.436
93	0	25.1	0	0	0	0	0	0	0	0	0	0	0	329	-554.45	-554.45	-0.0040	-0.385	-0.385
94	0	25.1	0	0	0	0	0	0	0	0	0	0	0	302	-554.45	-554.45	-0.0040	-0.385	-0.385
95	0	25.1	0	0	0	0	0	0	0	0	0	0	0	334	-554.45	-554.45	-0.0040	-0.385	-0.385
96	0	25.1	0	0	0	0	0	0	0	0	0	0	0	316	-554.45	-554.45	-0.0040	-0.385	-0.385
97	0	26.5	0	0	0	0	0	0	0	0	0	0	0	292	-551.98	-551.98	-0.0020	-0.188	-0.188
98	0	26.5	0	0	0	0	0	0	0	0	0	0	0	270	-551.98	-551.98	-0.0020	-0.188	-0.188
99	0	27.3	0	0	0	0	0	0	0	0	0	0	0	303	-550.57	-550.57	-0.0011	-0.103	-0.103
100	0	27.3	0	0	0	0	0	0	0	0	0	0	0	269	-550.57	-550.57	-0.0011	-0.103	-0.103
101	0	27.3	0	0	0	0	0	0	0	0	0	0	0	259	-550.57	-550.57	-0.0011	-0.103	-0.103

102	0	27.7	0	0	0	0	0	0	0	0	0	0	0	307	-549.86	-549.86	-0.0005	-0.053	-0.053
103	0	29.3	0	0	0	0	0	0	0	0	0	0	0	253	-547.04	-547.04	0.0015	0.148	0.148
104	0	29.3	0	0	0	0	0	0	0	0	0	0	0	281	-547.04	-547.04	0.0015	0.148	0.148
105	0	22.9	0	0	0	0	0	0	0	0	0	0	0	390	-558.33	-558.33	-0.0070	-0.669	-0.669
106	0	20.4	0	0	0	0	0	0	0	0	0	0	0	399	-562.92	-562.91	-0.0104	-0.998	-0.998
107	0	22.1	0	0	0	0	0	0	0	0	0	0	0	386	-559.74	-559.74	-0.0080	-0.770	-0.770
108	0	26.2	0	0	0	0	0	0	0	0	0	0	0	347	-552.33	-552.33	-0.0022	-0.213	-0.213
109	0	26.8	0	0	0	0	0	0	0	0	0	0	0	374	-551.28	-551.27	-0.0016	-0.154	-0.154
110	0	14.7	0	0	0	0.01	0	0	0	0	0	0	0	424	-573.15	-573.13	-0.0182	-1.746	-1.724
111	0	14.7	0	0	0	0.01	0	0	0	0	0	0	0	441	-573.15	-573.13	-0.0182	-1.746	-1.724
112	0	14.7	0	0	0	0.01	0	0	0	0	0	0	0	449	-573.15	-573.13	-0.0182	-1.746	-1.724
113	0	14.7	0	0	0	0.01	0	0	0	0	0	0	0	453	-573.15	-573.13	-0.0182	-1.746	-1.724
114	0	32.6	0	0	0	4.2	0	0	0	0	0	0	0	289	-504.32	-504.32	0.0059	0.564	0.564
115	0	25.6	0	0	0	1	0	0	0	0	0	0	0	339	-544.30	-544.30	-0.0020	-0.193	-0.193
116	0	28.4	0	0	0	0.99	0	0	0	0	0	0	0	280	-539.36	-539.36	0.0016	0.152	0.152
117	0	32.3	0	0	0	5	0	0	0	0	0	0	0	304	-498.16	-498.17	0.0052	0.495	0.495
118	0	17.4	0	0	0	4.5	0	0	0	0	0	0	0	448	-527.49	-527.47	-0.0118	-1.133	-1.133
119	0	17.5	0	0	0	1.9	0	0	0	0	0	0	0	436	-550.93	-550.91	-0.0118	-1.128	-1.128
120	0	19.5	0	0	0	2	0	0	0	0	0	0	0	420	-546.42	-546.41	-0.0091	-0.873	-0.873
121	0	20	0	0	0	4.1	0	0	0	0	0	0	0	434	-526.82	-526.81	-0.0086	-0.822	-0.822
122	0	22.2	0	0	0	4	0	0	0	0	0	0	0	410	-523.92	-523.91	-0.0058	-0.560	-0.560
123	0	24.2	0	0	0	1.9	0	0	0	0	0	0	0	399	-539.28	-539.28	-0.0032	-0.309	-0.309
124	0	24.5	0	0	0	4.2	0	0	0	0	0	0	0	396	-518.08	-518.07	-0.0032	-0.308	-0.308
125	0	25.3	0	0	0	6.4	0	0	0	0	0	0	0	379	-498.12	-498.12	-0.0044	-0.422	-0.422
126	0	22.7	0	0	0	1	0	0	0	0	0	0	0	386	-549.24	-549.23	-0.0057	-0.548	-0.548
127	0	29.5	0	0	0	5.2	0	0	0	0	0	0	0	343	-501.14	-501.14	0.0018	0.169	0.169
128	0	25.9	0	0	0	1.8	0	0	0	0	0	0	0	358	-536.11	-536.11	-0.0009	-0.090	-0.090
129	0	28.3	0	0	0	2.6	0	0	0	0	0	0	0	348	-525.37	-525.37	0.0022	0.208	0.208
130	0	30	0	0	0	2.7	0	0	0	0	0	0	0	318	-521.57	-521.57	0.0041	0.398	0.398
131	0	20.4	0	0	0	0.95	0	0	0	0	0	0	0	405	-554.45	-554.44	-0.0090	-0.864	-0.864
132	0	19.8	0	0	0	2.03	0	0	0	0	0	0	0	411	-545.09	-545.08	-0.0087	-0.835	-0.835
133	0	26.5	0	0	0	4.69	0	0	0	0	0	0	0	376	-510.98	-510.98	-0.0012	-0.120	-0.120
134	0	28.8	0	0	0	4.74	0	0	0	0	0	0	0	351	-506.12	-506.12	0.0013	0.128	0.128
135	0	22.9	0	0	0	6.1	0	0	0	0	0	0	0	402	-504.94	-504.94	-0.0067	-0.646	-0.646
136	0	24.4	0	0	0	6.4	0	0	0	0	0	0	0	379	-499.53	-499.53	-0.0053	-0.513	-0.513
137	0	27	0	0	0	5.9	0	0	0	0	0	0	0	363	-499.85	-499.85	-0.0018	-0.175	-0.175
138	0	29.5	0	0	0	5.8	0	0	0	0	0	0	0	335	-496.59	-496.59	0.0011	0.105	0.105
139	0	19.9	0	0	0	1.05	0	0	0	0	0	0	0	408	-554.18	-554.17	-0.0094	-0.899	-0.899
140	0	22.1	0	0	0	1	0	0	0	0	0	0	0	399	-550.30	-550.29	-0.0064	-0.613	-0.613
141	0	23.9	0	0	0	0.99	0	0	0	0	0	0	0	370	-547.12	-547.12	-0.0041	-0.396	-0.396
142	0	22.8	0	0	0	2.78	0	0	0	0	0	0	0	401	-533.29	-533.28	-0.0049	-0.467	-0.467
143	0	23.6	0	0	0	2.6	0	0	0	0	0	0	0	391	-533.48	-533.48	-0.0037	-0.350	-0.350
144	0	18.9	0	0	0	3.2	0	0	0	0	0	0	0	430	-536.07	-536.06	-0.0095	-0.910	-0.910
145	0	21.9	0	0	0	4.76	0	0	0	0	0	0	0	412	-517.76	-517.76	-0.0066	-0.635	-0.635
146	0.05	14	0	0	0	0	0	0	0	0	0	0	0	393	-574.21	-574.19	-0.0190	-1.821	-1.574
147	0.03	18.5	0	0	0	0	0	0	0	0	0	0	0	373	-566.09	-566.08	-0.0128	-1.229	-1.064
148	0.02	22.1	0	0	0	0	0	0	0	0	0	0	0	343	-559.74	-559.74	-0.0080	-0.770	-0.660
149	0.02	13.8	0	10	0	0	0	0	0	0	0	0	0	343	-541.69	-541.69	-0.0027	-0.259	-0.149
150	0.02	14.4	0	12.3	0	0	0	0	0	0	0	0	0	346	-533.11	-533.11	0.0007	0.066	0.176
151	0.02	13.9	0	13.7	0	0	0	0	0	0	0	0	0	311	-528.80	-528.80	0.0019	0.181	0.291
152	0.03	21.9	0	9.7	0	0	0	0	0	0	0	0	0	276	-528.48	-528.48	0.0055	0.530	0.694
153	0	16.6	0	0	0.3	0	0	0	0	0	0	0	0	395	-566.37	-566.36	-0.0128	-1.228	-1.228
154	0	15.9	0	0	0.72	0	0	0	0	0	0	0	0	363	-563.45	-563.44	-0.0104	-1.000	-1.000
155	0	17.2	0	0	1.23	0	0	0	0	0	0	0	0	330	-554.83	-554.83	-0.0039	-0.372	-0.372

156	0	16.1	0	0	0	0	0	0.97	0	0	0	0	0	415	-572.50	-572.49	-0.0149	-1.429	-1.429
157	0	17.3	0	0	0	0	0	2.01	0	0	0	0	0	416	-572.56	-572.55	-0.0124	-1.189	-1.189
158	0	17.2	0	0	0	0	0	3	0	0	0	0	0	419	-574.65	-574.64	-0.0119	-1.140	-1.140
159	0	17.1	0	0.9	0	0	0	0	0	0	0	0	0	416	-565.43	-565.42	-0.0130	-1.247	-1.247
160	0	17	0	1.89	0	0	0	0	0	0	0	0	0	401	-562.65	-562.64	-0.0119	-1.140	-1.140
161	0	17.2	0	2.9	0	0	0	0	0	0	0	0	0	393	-559.16	-559.15	-0.0096	-0.921	-0.921
162	0	17.1	0	0	0	0	0	0	1.04	0	0	0	0	365	-576.51	-576.50	-0.0096	-0.918	-0.918
163	0	17.3	0	0	0	0	0	0	2.09	0	0	0	0	333	-582.52	-582.51	-0.0053	-0.505	-0.505
164	0	17.2	0	0	0	0	0	0	3.08	0	0	0	0	284	-590.47	-590.47	-0.0003	-0.028	-0.028
165	0	17	0	0	0	0	1.54	0	0	0	0	0	0	386	-565.57	-565.56	-0.0100	-0.961	-0.961
166	0	16.9	0	0	0	0	3.08	0	0	0	0	0	0	364	-561.82	-561.82	-0.0040	-0.389	-0.389
167	0	16.6	0	0	0	0	4.46	0	0	0	0	0	0	341	-559.18	-559.18	0.0003	0.031	0.031
168	0	16.9	0	0	0	0	0	0	0	0.42	0	0	0	389	-568.00	-567.99	-0.0133	-1.277	-1.277
169	0	17	0	0	0	0	0	0	0	0.85	0	0	0	376	-566.17	-566.16	-0.0099	-0.955	-0.955
170	0	17.2	0	0	0	0	0	0	0	1.21	0	0	0	366	-564.55	-564.55	-0.0077	-0.740	-0.740
171	0	17.1	0.99	0	0	0	0	0	0	0	0	0	0	395	-570.48	-570.47	-0.0136	-1.310	-1.310
172	0	17.2	1.51	0	0	0	0	0	0	0	0	0	0	369	-572.40	-572.39	-0.0126	-1.207	-1.207
173	0	17.2	2.12	0	0	0	0	0	0	0	0	0	0	349	-573.36	-573.35	-0.0121	-1.161	-1.161
174	0	17.1	0	0	0	0.51	0	0	0	0	0	0	0	409	-564.02	-564.00	-0.0139	-1.337	-1.337
175	0	17.1	0	0	0	1	0	0	0	0	0	0	0	409	-559.12	-559.10	-0.0130	-1.247	-1.247
176	0	17	0	0	0	1.5	0	0	0	0	0	0	0	408	-554.57	-554.56	-0.0126	-1.208	-1.208
177	0	17.3	0	0	0	0	0	0	0	0	0.58	0	0	373	-565.22	-565.20	-0.0112	-1.076	-1.076
178	0	16.8	0	0	0	0	0	0	0	0	1.2	0	0	335	-562.28	-562.27	-0.0078	-0.747	-0.747
179	0	17.1	0	0	0	0	0	0	0	0	1.72	0	0	279	-558.93	-558.93	-0.0043	-0.416	-0.416
180	0	16.7	0	0	0	0	0	0	0	0	0	0.71	0	385	-565.89	-565.88	-0.0130	-1.246	-1.246
181	0	17.2	0	0	0	0	0	0	0	0	0	1.52	0	367	-561.81	-561.80	-0.0102	-0.979	-0.979
182	0	17.4	0	0	0	0	0	0	0	0	0	2.2	0	351	-558.09	-558.08	-0.0077	-0.738	-0.738
183	0	17	0	0	0	0	0	0	0	0	0	0	1.44	397	-567.01	-567.00	-0.0095	-0.911	-0.911
184	0	17	0	0	0	0	0	0	0	0	0	0	3.01	389	-564.34	-564.34	-0.0076	-0.731	-0.731
185	0	17.3	0	0	0	0	0	0	0	0	0	0	4.48	361	-562.09	-562.09	-0.0043	-0.413	-0.413
186	0.1	15.4	0	0	0	0	0	0	0	0	0	0	0	385	-571.74	-571.72	-0.0172	-1.648	-1.098
187	0.18	15.6	0	0	0	0	0	0	0	0	0	0	0	353	-571.38	-571.37	-0.0168	-1.616	-0.627
188	0.28	15.7	0	0	0	0	0	0	0	0	0	0	0	329	-571.38	-571.37	-0.0169	-1.622	-0.084
189	0	13.6	0	0	0	0	0	0	0	0	0	0	0	442	-574.91	-574.89	-0.0195	-1.871	-1.871
190	0	14.7	0	0	0	0	0	0	0	0	0	0	0	442	-572.80	-572.78	-0.0179	-1.721	-1.721
191	0	15.4	0	0	0	0	0	0	0	0	0	0	0	440	-571.74	-571.72	-0.0172	-1.648	-1.648
192	0	16	0	0	0	0	0	0	0	0	0	0	0	428	-570.68	-570.66	-0.0164	-1.571	-1.571
193	0	16.9	0	0	0	0	0	0	0	0	0	0	0	433	-569.27	-569.25	-0.0152	-1.462	-1.462
194	0	19.5	0	0	0	0	0	0	0	0	0	0	0	415	-564.33	-564.32	-0.0115	-1.102	-1.102
195	0	20	0	0	0	0	0	0	0	0	0	0	0	428	-563.62	-563.61	-0.0110	-1.056	-1.056
196	0	21.4	0	0	0	0	0	0	0	0	0	0	0	397	-561.15	-561.15	-0.0091	-0.873	-0.873
197	0	15.2	0	0	0	0	0	0	0	0	0	0	0	453	-572.09	-572.07	-0.0173	-1.664	-1.664
198	0	16.7	0	0	0	0	0	0	0	0	0	0	0	438	-569.27	-569.25	-0.0152	-1.462	-1.462
199	0	20.2	0	0	0	0	0	0	0	0	0	0	0	411	-563.27	-563.26	-0.0107	-1.031	-1.031
200	0.07	20.1	0.01	0	0	0.33	0	0	0	0	0	0	0	373	-560.68	-560.67	-0.0103	-0.991	-0.606
201	0.27	20	0.05	0	0	0.3	0	0	0	0	0	0	0	300	-561.04	-561.03	-0.0106	-1.017	0.467
202	0.08	19.4	0.11	0	0	1.86	0	0	0	0	0	0	0	383	-547.75	-547.74	-0.0094	-0.906	-0.467
203	0.06	20.5	0	0	0	0.1	0	0	0	0	0	0	0	373	-561.59	-561.58	-0.0105	-1.010	-0.681
204	0.06	20.1	0	2.2	0	0.1	0	0	0	0	0	0	0	363	-555.12	-555.12	-0.0067	-0.640	-0.311
205	0.04	19.8	0	5.8	0	0.1	0	0	0	0	0	0	0	313	-543.57	-543.56	-0.0012	-0.112	0.107
206	0.04	20.3	0	9.8	0	0.1	0	0	0	0	0	0	0	293	-529.69	-529.70	0.0042	0.406	0.626
207	0	15	5	9	0	5	0	0	0	0	0	0	0	304	-509.52	-509.52	0.0003	0.030	0.030
208	0.02	30.1	0	0	0	6.01	0	0	0	0	0	0	0	314	-493.57	-493.58	0.0016	0.153	0.241
209	0.01	15.7	0	0	0	0.01	0	0	0	0	0	0	0	447	-571.03	-571.02	-0.0166	-1.590	-1.557

210	0	24.4	0	0	0	0.02	0	0	0	0	0	0	0	390	-555.86	-555.86	-0.0051	-0.488	-0.477
211	0	28.1	0	0	0	5.9	0	0	0	0	0	0	0	343	-498.08	-498.08	-0.0007	-0.062	-0.062
212	0	32.3	0	0	0	6	0	0	0	0	0	0	0	293	-490.05	-490.05	0.0039	0.375	0.375
213	0	26	0	5	0	5.9	0	0	0	0	0	0	0	299	-485.94	-485.95	0.0053	0.511	0.511
214	0	28.6	0	5	0	6	0	0	0	0	0	0	0	293	-480.73	-480.73	0.0056	0.541	0.541
215	0	24.7	0	6.6	0	5.9	0	0	0	0	0	0	0	301	-483.04	-483.05	0.0031	0.302	0.302
216	0	17.5	5.5	8.5	0	5.1	0	0	0	0	0	0	0	254	-507.15	-507.15	0.0023	0.223	0.223
217	0	20.4	5	8	0	5	0	0	0	0	0	0	0	261	-503.48	-503.49	0.0044	0.425	0.425
218	0	22.2	5	8.2	0	4.9	0	0	0	0	0	0	0	256	-500.66	-500.67	0.0058	0.558	0.558
219	0	11.2	6.7	11.6	0	4.7	0	0	0	0	0	0	0	266	-514.50	-514.50	0.0006	0.058	0.058
220	0	16	4.9	11.6	0	5	0	0	0	0	0	0	0	267	-499.61	-499.61	0.0030	0.286	0.286
221	0	14.1	0	0	0	0.01	0	0	0	0	0	0	0	459	-574.21	-574.19	-0.0190	-1.821	-1.799
222	0.01	16.9	0	0	0	0.01	0	0	0	0	0	0	0	438	-568.92	-568.90	-0.0150	-1.436	-1.409
223	0	18.6	0	0	0	0.01	0	0	0	0	0	0	0	423	-566.09	-566.08	-0.0128	-1.229	-1.207
224	0.01	22.7	0	0	0	0.01	0	0	0	0	0	0	0	371	-558.68	-558.68	-0.0072	-0.693	-0.665
225	0	25	0	0	0	0	0	5	0	0	0	0	0	347	-564.89	-564.89	-0.0031	-0.296	-0.296
226	0	25	0	0	0	0	0	8	0	0	0	0	0	353	-570.98	-570.98	-0.0027	-0.261	-0.261
227	0	30	0	0	0	0	0	5	0	0	0	0	0	310	-556.07	-556.07	0.0015	0.147	0.147
228	0	30	0	0	0	0	0	8	0	0	0	0	0	310	-562.16	-562.16	0.0011	0.105	0.105
229	0	24.3	0	0	0	3	0	0	0	0	0	0	0	396	-528.86	-528.85	-0.0030	-0.290	-0.290
230	0	26	0	0	0	3.7	0	0	0	0	0	0	0	385	-520.16	-520.15	-0.0011	-0.104	-0.104
231	0	26	0	0	0	3.7	0	0	0	0	0	0	0	390	-520.16	-520.15	-0.0011	-0.104	-0.104
232	0	27.3	0	0	0	3.4	0	0	0	0	0	0	0	376	-519.65	-519.65	0.0007	0.066	0.066
233	0	27.9	0	0	0	2.6	0	0	0	0	0	0	0	362	-526.07	-526.08	0.0017	0.160	0.160
234	0	27.8	0	0	0	3.2	0	0	0	0	0	0	0	375	-520.90	-520.90	0.0012	0.114	0.114
235	0	30.3	0	0	0	4.7	0	0	0	0	0	0	0	308	-504.63	-504.63	0.0030	0.293	0.293
236	0	32	0	0	0	3.9	0	0	0	0	0	0	0	298	-507.96	-507.97	0.0058	0.559	0.559
237	0	17.9	0	0	0	0	0	5.1	0	0	0	0	0	383	-577.59	-577.58	-0.0096	-0.921	-0.921
238	0	18.1	0	0	0	0	0	3	0	0	0	0	0	412	-572.89	-572.88	-0.0108	-1.036	-1.036
239	0	17.9	0	0	0	0	0	0.8	0	0	0	0	0	416	-568.89	-568.88	-0.0128	-1.228	-1.228
240	0	21.1	0	0	0	0	0	5	0	0	0	0	0	392	-571.95	-571.94	-0.0066	-0.634	-0.634
241	0	20.6	0	0	0	0	0	3	0	0	0	0	0	394	-568.30	-568.29	-0.0079	-0.760	-0.760
242	0	21.4	0	0	0	0	0	1.1	0	0	0	0	0	403	-563.33	-563.32	-0.0083	-0.797	-0.797
243	0	24.8	0	0	0	0	0	2.9	0	0	0	0	0	357	-560.89	-560.89	-0.0035	-0.332	-0.332
244	0	25.1	0	0	0	0	0	1	0	0	0	0	0	352	-556.63	-556.62	-0.0037	-0.352	-0.352
245	0	29.3	0	0	0	0	0	5.3	0	0	0	0	0	292	-557.56	-557.56	0.0010	0.096	0.096
246	0	29.1	0	0	0	0	0	1	0	0	0	0	0	285	-549.57	-549.57	0.0012	0.113	0.113
247	0	29.8	0	0	0	0	0	5.1	0	0	0	0	0	274	-556.42	-556.42	0.0014	0.132	0.132
248	0	30	0	0	0	0	0	5.1	0	0	0	0	0	260	-556.07	-556.07	0.0015	0.147	0.147
249	0	30.3	0	0	0	0	0	5.5	0	0	0	0	0	285	-556.23	-556.24	0.0018	0.175	0.175
250	0	31	0	0	0	0	0	6	0	0	0	0	0	280	-556.05	-556.05	0.0025	0.237	0.237
251	0	16.1	0	0	0	1.7	0	0	0	0	0	0	0	435	-554.38	-554.36	-0.0136	-1.309	-1.309
252	0	19	0	0	0	2.6	0	0	0	0	0	0	0	448	-541.60	-541.59	-0.0094	-0.904	-0.904
253	0	19.5	0	0	0	1.6	0	0	0	0	0	0	0	420	-549.36	-549.35	-0.0091	-0.877	-0.877
254	0	20	0	0	0	3.1	0	0	0	0	0	0	0	430	-535.29	-535.28	-0.0083	-0.797	-0.797
255	0	14.8	0	0	0	0	0	0	0	0	0	0	0	467	-572.80	-572.78	-0.0179	-1.721	-1.721
256	0	24.7	0	0	0	0	0	0	0	0	0	0	0	291	-555.16	-555.15	-0.0045	-0.436	-0.436
257	0	21	0	0	0	0	0	0	0	0	0	0	0	406	-561.86	-561.85	-0.0097	-0.928	-0.928
258	0.02	31.6	0	0	0	6.45	0	0	0	0	0	0	0	273	-487.54	-487.54	0.0025	0.243	0.342
259	0.02	18.5	5.03	7.96	0	4.75	0.15	0	0	0	0	0	0	253	-508.62	-508.62	0.0029	0.274	0.362
260	0	20	0	7	0	6	0	0	1	0	0	0	0	300	-495.29	-495.29	0.0014	0.138	0.138
261	0	16.3	0	0	0	5.73	0	0	0	0	0	0	0	425	-518.82	-518.81	-0.0137	-1.317	-1.306
262	0.22	17.2	0	0	0	0.04	0	0	0	0	0	0	0	339	-568.92	-568.90	-0.0150	-1.436	-0.227
263	0.27	17.1	0	0	0	0.04	0	0	0	0	0	0	0	300	-568.92	-568.90	-0.0150	-1.436	0.047

264	0.01	30.7	0	0	0	4.95	0	0	0	0	0	0	0	293	-500.99	-500.99	0.0033	0.321	0.359
265	0	26.4	0	5.2	0	6.2	0	0	0	0	0	0	0	307	-482.02	-482.02	0.0034	0.325	0.325
266	0	26.4	0	5.2	0	6.2	0	0	0	0	0	0	0	311	-482.02	-482.02	0.0034	0.325	0.325
267	0	26.4	0	5.2	0	6.2	0	0	0	0	0	0	0	311	-482.02	-482.02	0.0034	0.325	0.325
268	0	26.4	0	5.2	0	6.2	0	0	0	0	0	0	0	313	-482.02	-482.02	0.0034	0.325	0.325
269	0	26.4	0	5.2	0	6.2	0	0	0	0	0	0	0	313	-482.02	-482.02	0.0034	0.325	0.325
270	0	20	4.7	8.1	0	5.1	0	0	0	0	0	0	0	257	-503.21	-503.21	0.0040	0.383	0.383
271	0	29.9	0	0	0	6	0	0	0	0	0	0	0	307	-493.93	-493.93	0.0014	0.131	0.131
272	0	26.4	0	5.2	0	6.02	0	0	0	0	0	0	0	323	-483.63	-483.63	0.0037	0.356	0.356
273	0	28.1	0	5.2	0	6.1	0	0	0	0	0	0	0	275	-479.82	-479.83	0.0039	0.374	0.374
274	0	30	0	5.3	0	6.1	0	0	0	0	0	0	0	268	-476.02	-476.03	0.0068	0.656	0.656
275	0	30.3	0	0	0	6.1	0	0	0	0	0	0	0	330	-492.24	-492.24	0.0017	0.166	0.166
276	0.04	28.4	0	0	0	6.2	0	0	0	0	0	0	0	297	-494.79	-494.79	-0.0007	-0.068	0.124
277	0.01	13	5.6	10	0	4.9	0	0	0	0	0	0	0	281	-512.46	-512.46	0.0002	0.020	0.075
278	0.01	20.4	0	7.3	0	5.6	0	0	0.97	0	0	0	0	299	-496.89	-496.90	0.0029	0.281	0.309
279	0	30.3	0	0	0	6.1	0	0	0	0	0	0	0	300	-492.24	-492.24	0.0017	0.166	0.166
280	0	26.4	0	5.2	0	6	0	0	0	0	0	0	0	315	-483.63	-483.63	0.0037	0.356	0.356
281	0	17.8	4.1	7.8	0	4.7	0	0	0	0	0	0	0	263	-509.36	-509.37	0.0017	0.165	0.165
282	0	17.8	4.1	7.8	0	4.7	0	0	0	0	0	0	0	282	-509.36	-509.37	0.0017	0.165	0.165
283	0	17.8	4.1	7.8	0	4.7	0	0	0	0	0	0	0	312	-509.36	-509.37	0.0017	0.165	0.165
284	0	17.8	4.1	7.8	0	4.7	0	0	0	0	0	0	0	321	-509.36	-509.37	0.0017	0.165	0.165
285	0	30.3	0	0	0	6.1	0	0	0	0	0	0	0	329	-492.24	-492.24	0.0017	0.166	0.166
286	0	14.6	4.1	8	0	4.2	0	0	0	0	0	0	0	304	-517.95	-517.95	-0.0009	-0.082	-0.082
287	0.05	25.4	0	7.4	0	6.1	0	0	1.1	0	0	0	0	279	-485.47	-485.48	0.0080	0.767	1.053
288	0.05	20.6	0	9.3	0	5.83	0	0	1	0	0	0	0	285	-488.66	-488.67	0.0052	0.499	0.746
289	0.03	19.6	0	7.3	0	6.15	0	0	0.97	0	0	0	0	291	-493.76	-493.76	0.0011	0.105	0.253
290	0	15.5	4.98	9.11	0	7.03	0	0	0	0	0	0	0	297	-492.15	-492.15	-0.0010	-0.092	-0.092
291	0.16	19.6	0	0	0	0.05	0	0	0	0	0	0	0	322	-564.33	-564.32	-0.0115	-1.102	-0.223
292	0.04	23.6	0	0	0	0.05	0	0	0	0	0	0	0	333	-557.27	-557.27	-0.0062	-0.592	-0.372
293	0.19	24.1	0	0	0	0.02	0	0	0	0	0	0	0	280	-556.57	-556.56	-0.0056	-0.540	0.504
294	0.02	24.2	0	0	0	0.02	0	0	0	0	0	0	0	377	-556.22	-556.21	-0.0054	-0.514	-0.405
295	0.14	25	0	0	0	0.05	0	0	0	0	0	0	0	267	-554.80	-554.80	-0.0043	-0.410	0.359
296	0.16	19.3	0	0	1.5	0.05	0	0	0	0	0	0	0	245	-549.13	-549.13	0.0004	0.037	0.916
297	0.15	25.6	0	0	0.57	0.16	0	0	0	0	0	0	0	256	-545.64	-545.64	0.0018	0.174	0.998
298	0.16	24.8	0	0	0.79	0.05	0	0	0	0	0	0	0	236	-546.84	-546.84	0.0021	0.204	1.083
299	0.15	24.9	0	0	2.22	0.05	0	0	0	0	0	0	0	167	-532.38	-532.39	0.0123	1.177	2.001
300	0.18	23.3	0	3.21	0	0.06	0	0	0	0	0	0	0	253	-546.34	-546.34	-0.0010	-0.097	0.892
301	0.18	22.8	0	5.34	0	0.07	0	0	0	0	0	0	0	233	-540.51	-540.51	0.0013	0.126	1.115
302	0.02	27.8	0	0	0	4.1	0	0	0	0	0	0	0	327	-513.41	-513.41	0.0007	0.070	0.180
303	0.02	31.8	0	0	0	5.22	0	0	0	0	0	0	0	284	-497.26	-497.26	0.0043	0.412	0.522
304	0.02	32	0	0	0	6.1	0	0	0	0	0	0	0	264	-489.42	-489.42	0.0036	0.345	0.455
305	0.02	31.6	0	2.06	0	6.19	0	0	0	0	0	0	0	290	-483.23	-483.23	0.0052	0.501	0.611
306	0.18	15.2	4.16	7.92	0	4.78	0	0	0	0	0	0	0	235	-512.97	-512.97	-0.0010	-0.094	0.895
307	0	20.5	0	8.9	0	5.9	0	0	0	0	0	0	0	311	-482.58	-482.58	0.0023	0.221	0.221
308	0	16.4	4.03	9.2	0	5.13	0	0	0	0	0	0	0	346	-503.53	-503.53	0.0020	0.193	0.193
309	0	30.3	0	0	0	5.8	0	0	0	0	0	0	0	300	-495.18	-495.18	0.0020	0.189	0.189
310	0	14.3	4.9	9.2	0	5.1	0	0	0	0	0	0	0	290	-508.97	-508.97	-0.0002	-0.018	-0.018
311	0	15.3	5	7.5	0	5.3	0	0	0	0	0.6	0	0	203	-508.25	-508.25	0.0013	0.121	0.121
312	0	30	2.5	5	0	7	0	0	0	0	0	0	0	300	-475.54	-475.55	0.0066	0.631	0.631
313	0	30	0	0	0	5	0	4	0	0	0	0	0	300	-509.87	-509.88	0.0033	0.317	0.317
314	0	29	2	4.5	0	5.5	0	3.5	0	0	0	0	0	300	-495.64	-495.65	0.0058	0.561	0.561
315	0	13.7	4.9	8.3	0	6	0	0	0	0	0	0	0	340	-505.68	-505.68	-0.0026	-0.245	-0.245
316	0	24	0	0	0	0	0	0	0	0	0	0	0	367	-556.22	-556.21	-0.0054	-0.514	-0.505
317	0	24.3	0	0	0	5.88	0	0	0	0	0	0	0	376	-504.43	-504.43	-0.0049	-0.471	-0.456

318	0	15.8	0	0	0	0	0	0	0	0	0	0	0	410	-571.03	-571.02	-0.0166	-1.597	-1.597
319	0	15.6	0	0	0	2.7	0	0	0	0	0	0	0	437	-546.61	-546.60	-0.0137	-1.314	-1.314
320	0	16	0	0	0	5.4	0	0	0	0	0	0	0	422	-522.11	-522.10	-0.0138	-1.323	-1.323
321	0	25.3	0	0	0	2.8	0	0	0	0	0	0	0	343	-528.70	-528.70	-0.0015	-0.147	-0.147
322	0	25	0	0	0	5.2	0	0	0	0	0	0	0	341	-508.90	-508.90	-0.0035	-0.335	-0.335
